# REGγ is essential to maintain bone homeostasis by degrading TRAF6, preventing osteoporosis

**DOI:** 10.1073/pnas.2405265121

**Published:** 2024-11-13

**Authors:** Yingying Du, Hui Chen, Lei Zhou, Qunfeng Guo, Shuangming Gong, Siyuan Feng, Qiujing Guan, Peilin Shi, Tongxin Lv, Yilan Guo, Cheng Yang, Peng Sun, Kun Li, Shuogui Xu, Lei Li

**Affiliations:** ^a^Shanghai Key Laboratory of Regulatory Biology, Institute of Biomedical Sciences, School of Life Sciences, East China Normal University, Shanghai 200241, China; ^b^Department of Trauma-Emergency and Critical Care Medicine, Shanghai Fifth People’s Hospital, Fudan University, Shanghai 200240, China; ^c^Joint Center for Translational Medicine, Shanghai Fifth People’s Hospital, Fudan University and School of Life Science, East China Normal University, Shanghai 200241, China; ^d^School of Life Sciences, East China Normal University, Shanghai 200241, China; ^e^Department of Orthopedics, Shanghai General Hospital, Shanghai Jiao Tong University School of Medicine, Shanghai 200080, China; ^f^Department of Orthopedics, Changzheng Hospital, Naval Medical University, Shanghai 200003, China; ^g^The Key Laboratory of Adolescent Health Assessment and Exercise Intervention of the Ministry of Education, East China Normal University, Shanghai 200241, China; ^h^Health Science Center, East China Normal University, Shanghai 200241, China; ^i^Department of Emergency and Trauma, The First Affiliated Hospital of Naval Medical University, Shanghai 200433, China; ^j^Chongqing Key Laboratory of Precision Optics, Chongqing Institute of East China Normal University, Chongqing 401120, China; ^k^East China Normal University, Shanghai Frontiers Science Center of Genome Editing and Cell Therapy, Shanghai 200241, China

**Keywords:** ubiquitin-independent degradation, REGγ, TRAF6, NIP30, osteoporosis

## Abstract

Primary osteoporosis, manifesting as decreased bone mass and increased bone fragility, is a “silent disease” that is often ignored until a bone breaks. Accordingly, it is urgent to develop reliable biomarkers and novel therapeutic strategies for osteoporosis treatment. Here, we identified REGγ as a potential biomarker of osteoporotic populations through proteomics analysis. Next, we demonstrated that REGγ deficiency increased osteoclast activity and triggered bone mass loss in *REGγ* knockout (KO) and bone marrow-derive macrophage (BMM)-conditional *REGγ* KO mice. However, the osteoclast activity decreased in BMM-conditional *REGγ* overexpression mice. Mechanistically, we defined that REGγ-20S proteasome directly degraded TRAF6 to inhibit bone absorption in a ubiquitin-independent pathway. More importantly, BMM-conditional *Traf6* KO with *REGγ* KO mice could “rescue” the osteoporosis phenotypes. Based on NIP30 (a REGγ “inhibitor”) dephosphorylation by CKII inhibition activated the ubiquitin-independent degradation of TRAF6, we selected TTP22, an inhibitor of CKII, and defined that TTP22 could alleviate osteoporosis in vitro and in vivo. Overall, our study reveals a unique function of NIP30/REGγ/TRAF6 axis in osteoporosis and provides a potential therapeutic drug TTP22 for osteoporosis.

## Significance

Primary osteoporosis torments numerous older adults, which is a common systemic bone disease. Based on the potential pathogenesis of osteoporosis by proteomics analysis for osteoporosis samples and experiments in biochemistry and molecular biology, we demonstrated that TRAF6 could be degraded by REGγ via ubiquitin/ATP (Adenosine Triphosphate)-independent degradation manner directly and the dephosphorylation of NIP30 modulated by the inhibitor of CKII TTP22 promoted the degradation of TRAF6 by REGγ-20S proteasome to postpone osteoporosis. Based on the pivotal mechanism of the NIP30/REGγ/TRAF6 axis, we defined that the CKII inhibitor TTP22 could alleviate osteoporosis by promoting the ubiquitin-independent proteasomal degradation of TRAF6 and provided a strategy for the treatment of osteoporosis.

Primary osteoporosis, which affects numerous older adults, especially postmenopausal women, is a common systemic bone disease that mainly manifests in decreased bone mass and increased bone fragility, which could lead to bone microstructure degradation, such as low back pain and spinal deformation, and fragile fractures (1–6). Furthermore, fragile fractures secondary to osteoporosis are a major concern because of their frequency and health hazards (7–9). The silent progression of osteoporosis disrupts early diagnosis and effective treatment. Bone mineral density (BMD), as the diagnostic gold standard, gives partial information on bone strength (10). Hence, complementary methods, such as biomarkers, are essential for early diagnosis. However, the existing biomarkers are not accurate for early diagnosis (11, 12). Current pharmacologic therapies, such as RANKL (Receptor Activator of Nuclear Factor Kappa-B Ligand) antibodies and parathyroid hormone-related peptide analogs, have only reduced fragile fractures to some extent (7, 13–16). At present, bisphosphonate and hormone therapy are the commonly used, but bisphosphonate is not easily absorbed by the human body and causes adverse reaction to the digestive tract. Hormone therapy tends to increase the incidence rate of other diseases, such as cancer and cardiovascular disease. Therefore, doubts about the long-term efficacy and adverse events of these drugs have prevented individuals from adhering to antiosteoporosis therapy (17, 18). Accordingly, it is still urgent to explore the underlying mechanisms of osteoporosis to develop reliable biomarkers and novel agents.

Enhanced osteoclastic bone resorption is one of the principal causes and therapeutic targets of osteoporosis (19). RANKL plays a key regulatory role in osteoclast maturation and activation (20–22). The binding of RANKL to RANK recruits the crucial adaptor molecule tumor necrosis factor receptor (TNFR)-associated factor 6 (TRAF6) to orchestrate subsequent signaling, including the NF-κβ, AP-1, and MAPK pathways, activating NFATC1 and resulting in excessive activation of osteoclasts (23–26). *Traf6* knockout (KO) in mice resulted in osteoclast deficiency and osteopetrosis (27), which indicates that the regulation of TRAF6 could be the direction for osteoporosis therapy. Recent studies found that the proteasome system could regulate the function of TRAF6 via degradation (28, 29), indicating that understanding the regulation of TRAF6 degradation could be a new therapeutic strategy for osteoporosis. However, to our knowledge, whether the ubiquitin-independent proteasome participates in TRAF6 turnover and osteoclastogenesis remains unknown.

REGγ, also named Ki antigen, PSME3, PA28γ, or 11Sγ, was first found in the serum of patients with systemic lupus erythematosus and could regulate proteolysis in a ubiquitin-independent pathway by activating the 20S proteasome (30, 31). *REGγ* KO cells display retarded growth, decreased proliferation, and increased apoptosis (32, 33). Our previous research also found that *REGγ*-deficient mice exhibited an aging phenotype and osteoporosis symptoms (34). In addition, NIP30, also known as PIP30, attenuates REGγ-20S proteasome activation through its direct binding to REGγ. The evolutionarily conserved serine-rich domain in the C-terminus of NIP30 is a posttranslational modification site that can be phosphorylated by CKII to enhance its inhibitory binding to REGγ (35, 36). Hence, we propose that CKII inhibition could be the strategy for augmenting the function of the REGγ-20S proteasome.

In this study, we identified REGγ as a candidate biomarker in osteoporosis and demonstrated that REGγ regulated bone metabolism by degrading TRAF6 in a ubiquitin-independent manner. Furthermore, we found that TTP22 could postpone osteoporosis by regulating theCKII/NIP30/REGγ/TRAF6 axis and could thereby provide a therapeutic approach for osteoporosis treatment. Collectively, our study reveals that the NIP30/REGγ/TRAF6 axis is critical in osteoporosis and TTP22 is a potential unique drug for osteoporosis treatment.

## Results

### REGγ Is Identified as a Potential Biomarker in Osteoporosis.

**Fig. 1.**
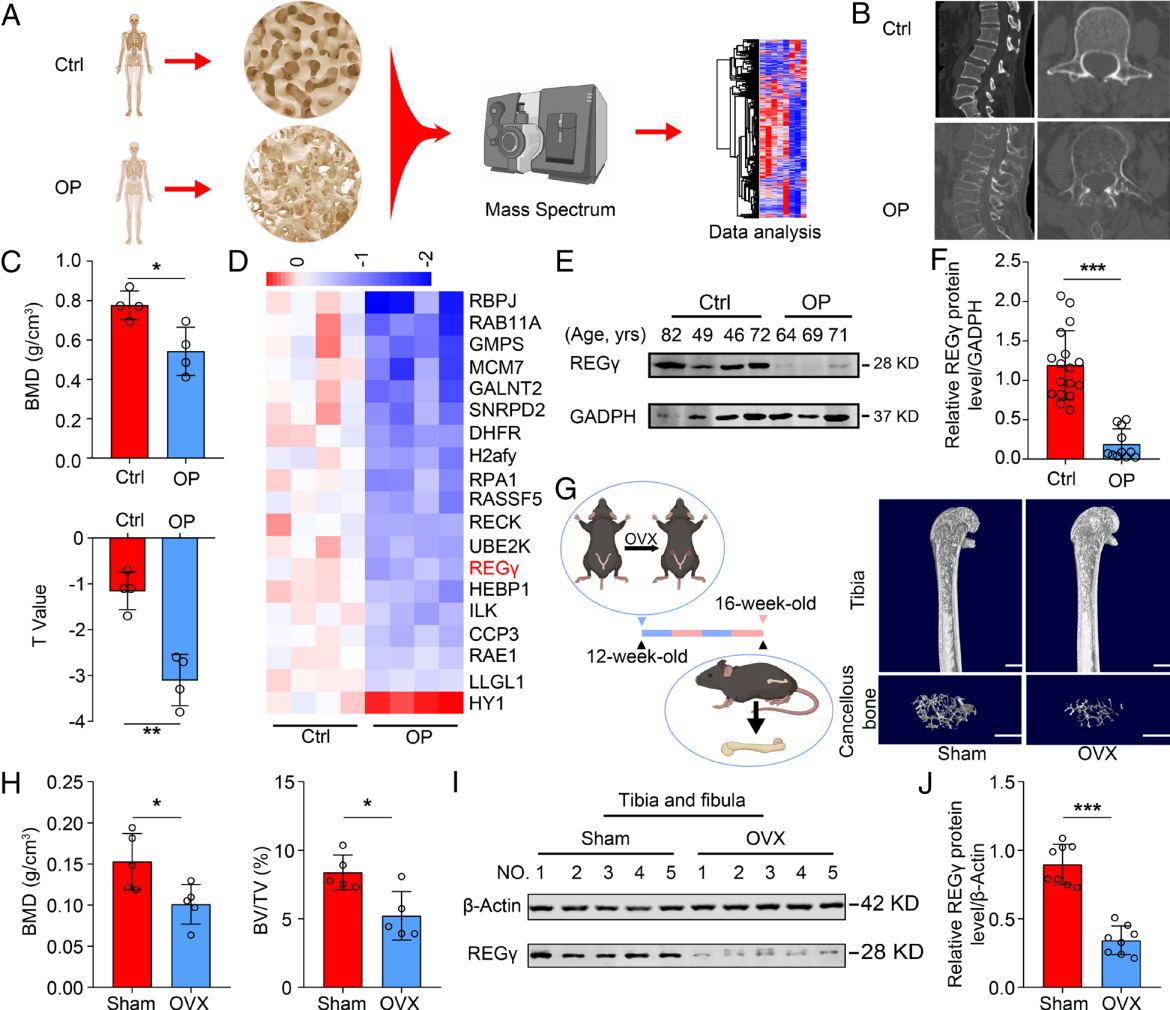
REGγ is identified as a potential biomarker in osteoporosis. (*A*) Workflow for the discovery of differentially expressed protein proteins by MS using control and osteoporosis (OP) lumbar cancellous bone tissues. (*B*) Representative spine CT images of Ctrl and OP patients. (*C*) Quantitative analysis of the BMD and T values of Ctrl and OP patients (n = 4). BMD, bone mineral density. (*D*) Heatmap of the differentially expressed proteins in Ctrl and OP samples. (*E* and *F*) Western blotting analysis of REGγ protein levels in Ctrl and OP samples (*E*), with quantification using ImageJ (*F*) (Ctrl n = 17, OP n = 11). (*G*) Schematic diagram of the OVX-induced osteoporosis mouse model (*Left*), with representative micro-CT images displaying the 3D bone structures of femurs from 4-mo-old sham and OVX mice (similar results were obtained in all mice, n = 5). (Scale bar: 1 mm.) (*H*) Micro-CT measurements of BMD and BV/TV in femurs from 4-mo-old sham and OVX mice. BV/TV, bone volume as a fraction of total bone volume (n = 5). (*I* and *J*) Western blotting analysis of REGγ protein levels in sham and OVX mice (*I*), with quantification using ImageJ (*J*) (n = 8). Markers of significance are as follows: N.S, *P* > 0.05; *P < 0.05; **P < 0.01; ***P < 0.001. Control: Ctrl; osteoporosis: OP. Sham: Sham operation; OVX: ovariectomy.

To investigate candidate biomarkers of osteoporosis, we collected osteoporosis and control samples from patients and measured the BMD by dual-energy X-ray absorptiometry (DXA) (Fig. 1 *A*–*C* and *SI Appendix*, Table S1). Through proteomic analysis of trabecular bone specimens from the above groups, we found 4,015 proteins, including 536 downregulated proteins and 69 upregulated proteins (*SI Appendix*, Fig. S1*A*), in the first proteomic analysis of osteoporosis samples from patients. Screening the differentially expressed proteins (OP/Ctrl ratio ≥ 1.2 or ≤ 0.83, *P* value ≤ 0.001) (Fig. 1*D*) demonstrated that REGγ was markedly downregulated in osteoporosis patients. Interestingly, our previous study reported that the deficiency of REGγ could promote premature aging (34). However, it was noteworthy that the downregulation of REGγ in osteoporosis was independent of age, and its expression levels positively correlated with bone mineral density (BMD) (Fig. 1 *E* and *F* and *SI Appendix*, Fig. S1*B*). Moreover, we observed the same phenomenon in serum samples from both healthy individuals and patients (*SI Appendix*, Fig. S1 *C* and *D*). This suggests that REGγ might be a potential biomarker for osteoporosis. Next, we generated an ovariectomy (OVX)-induced osteoporosis mouse model and found lower trabecular BMD and bone volume (BV/TV) in the OVX group than in the sham control group (Fig. 1 *G* and *H*). Similarly, the expression of REGγ in the OVX group was lower than that in the sham control group (Fig. 1 *I* and *J* and *SI Appendix*, Fig. S1*E*). These observations also indicated that REGγ is a potential biomarker in osteoporosis.

### Deficiency of REGγ Triggers the Loss of Bone Mass by Enhancing the Activity of Osteoclasts.

**Fig. 2.**
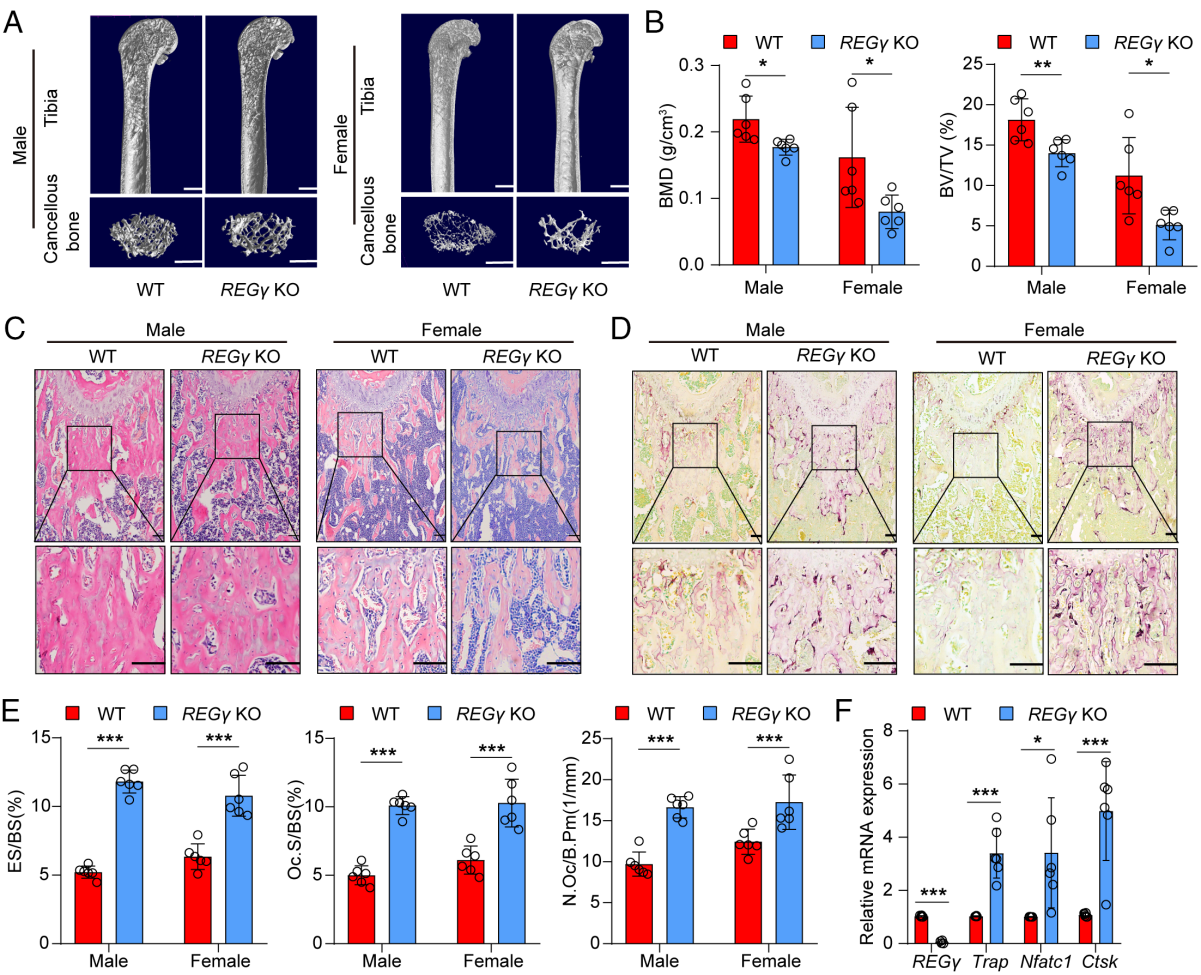
Deficiency of REGγ triggers the loss of bone mass by enhancing the activity of osteoclasts. (*A*) Representative micro-CT images showing the 3D bone structures of femurs from 6-mo-old WT littermates and *REGγ* KO mice (n = 6). (Scale bar: 1 mm.) (*B*) Micro-CT measurements of BMD and BV/TV in femurs from 6-mo-old WT littermates and *REGγ* KO mice (n = 6). (*C*) Representative images of H&E staining of femurs of 6-mo-old *REGγ* KO mice and their WT littermates (n = 6). (Scale bar: 100 μm.) (*D*) Representative immunofluorescence images of TRAP expression in 6-mo-old WT littermates and *REGγ* KO mice (n = 6). (Scale bar: 100 μm.) (*E*) Histomorphometrical analysis of TRAP staining in (*D*) (n = 6). (*F*) Quantification of *Trap*, *Nfatc1*, and *Ctsk* expression in femurs of 6-mo-old *REGγ* KO mice and WT littermates (n = 6). Markers of significance are as follows: N.S, *P* > 0.05; *P < 0.05; **P < 0.01; ***P < 0.001. WT: wildtype; KO: knockout.

Bone homeostasis is maintained by coordinated cycles of osteoclast bone resorption and osteoblast bone formation (18, 37–39). To further explore the function of REGγ in bone homeostasis, we analyzed the bone mass of wild-type (WT) and *REGγ* KO mice. The results showed that *REGγ* KO mice displayed bone loss compared to WT mice, as evidenced by the trabecular BMD and BV/TV (Fig. 2 *A* and *B*). However, the cortical BMD and BV/TV were not significantly different between WT and *REGγ* KO mice (*SI Appendix*, Fig. S2 *A* and *B*). Moreover, the Hematoxylin and Eosin (H&E) staining assay revealed the same results in the trabeculae of *REGγ* KO mice (Fig. 2*C* and *SI Appendix*, Fig. S2*C*). Taken together, these data demonstrated that REGγ deficiency leads to low bone mass.

To investigate whether the loss of bone mass in *REGγ* KO mice is caused by increased bone resorption or decreased bone formation, we detected the osteoclast marker tartrate-resistant acid phosphatase (TRAP) and the osteoblast marker osteocalcin (OCN). We found the presence of an increasing number of osteoclasts in the trabecular bone of *REGγ* KO mice (Fig. 2 *D* and *E*), whereas there was no difference in OCN levels between WT and *REGγ* KO mice (*SI Appendix*, Fig. S2 *D* and *E*). Subsequently, osteoclast markers, including *Trap*, *Nfatc1*, and *Ctsk*, were significantly increased in femoral samples from *REGγ*-deficient mice (Fig. 2*F*). In contrast, osteogenic markers showed no significant differences (*SI Appendix*, Fig. S2*F*), and REGγ deficiency did not affect osteoclast-to-osteoblast coupling factors (*SI Appendix*, Fig. S2*G*).

To further assess the role of REGγ in bone resorption and bone formation, we isolated bone marrow–derived macrophages (BMMs) and mesenchymal stem cells (MSCs) from both WT and *REGγ* KO mice. BMMs and MSCs were utilized for osteoclast differentiation (*SI Appendix*, Fig. S2*H*) and osteoblast differentiation (*SI Appendix*, Fig. S2*K*), respectively. Ex vivo osteoclast differentiation assay revealed an increase osteoclast formation of *REGγ* KO mice compared with that of WT mice (*SI Appendix*, Fig. S2 *I* and *J*). However, ALP staining, Von Kossa staining, and Alizarin Red S staining indicated no significant difference in osteoblast differentiation between WT and *REGγ* KO MSCs at various stages (*SI Appendix*, Fig. S2*L*). Additionally, qRT-PCR results demonstrated no significant changes in osteoblast markers, including *Alp*, *Osteocalcin* (*Ocn*), and *Osterix* (*SI Appendix*, Fig. S2*M*). Interestingly, we found that the expression of REGγ gradually decreases during osteoclast differentiation, and this reduction largely depends on Nrf2. (*SI Appendix*, Fig. S2 *N–Q*). Collectively, these results suggest that REGγ deficiency triggers the loss of bone mass by enhancing the activity of osteoclasts.

### REGγ Specifically Suppresses Osteoclast Activity.

**Fig. 3.**
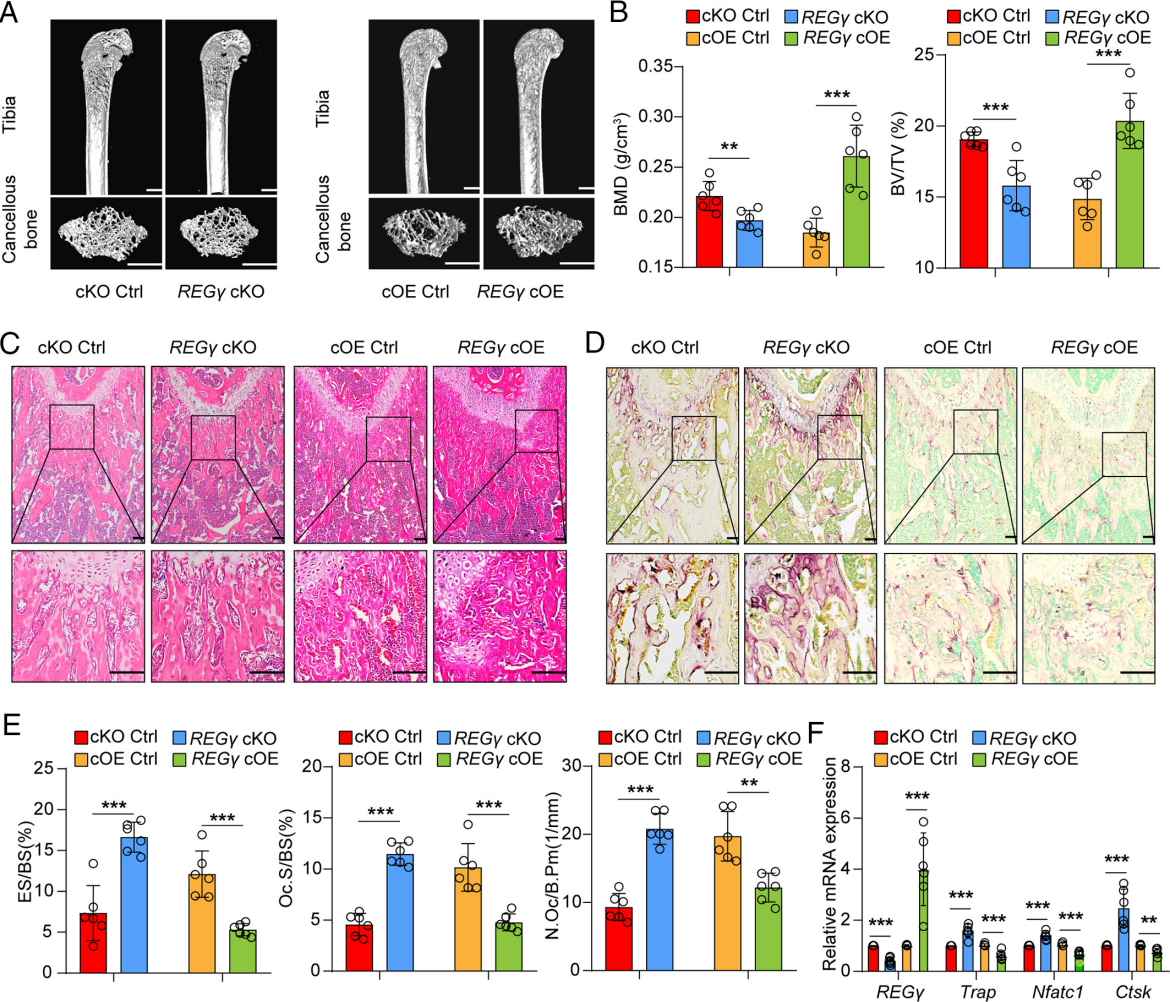
REGγ specifically suppressed osteoclast activity. (*A*) Representative micro-CT images showing the 3D bone structures of femurs from 2-mo-old *REGγ*^fl/fl^ (control), *REGγ* cKO, LSL-*REGγ* (control), and *REGγ* cOE mice (n = 6). (Scale bar: 1 mm.) (*B*) Micro-CT measurements of BMD and BV/TV in femurs from 2-mo-old cKO Ctrl, *REGγ* cKO, cOE Ctrl, and *REGγ* cOE mice (n = 6). (*C*) Representative images of H&E staining of femurs of 2-mo-old cKO Ctrl, *REGγ* cKO, cOE Ctrl, and *REGγ* cOE mice (n = 6). (Scale bar: 100 μm.) (*D*) Representative TRAP staining of femurs from 2-mo-old cKO Ctrl, *REGγ* cKO, cOE Ctrl, and *REGγ* cOE mice. (Scale bar: 1 mm.) (*E*) Histomorphometrical analysis of TRAP staining in (*E*) (n = 6). (*F*) Quantification of *Trap*, *Nfatc1*, and *Ctsk* expression in femurs of 2-mo-old cKO Ctrl, *REGγ* cKO, cOE Ctrl, and *REGγ* cOE mice (n = 6). Markers of significance are as follows: N.S, *P* > 0.05; *P < 0.05; **P < 0.01; ***P < 0.001. Control: Ctrl; *REGγ* cKO: bone marrow-derive macrophage (BMM)-conditional *REGγ* KO; *REGγ* cOE: bone marrow-derive macrophage (BMM)-conditional *REGγ* OE.

To verify whether REGγ deficiency triggers the loss of bone mass in an osteoclast-specific manner, we generated BMM-conditional *REGγ*-KO (*REGγ* cKO) mice and *REGγ*-overexpressing (*REGγ* cOE) mice by crossing *LysM*-Cre mice with floxed alleles of *REGγ*^fl/fl^ and LSL-*REGγ* mice, respectively (40, 41). We successfully obtained *REGγ* cKO and cOE mice. Both *REGγ* cKO and *REGγ* cOE mice showed no changes in size and weight compared with their controls at 2 mo (*SI Appendix*, Fig. S3 *A*, *B*, *F*, and *G*). However, the trabecular bone mass of the *REGγ* cKO mice decreased compared to that of the controls, while the trabecular bone mass of *REGγ* cOE mice showed the opposite phenomenon, as evidenced by an obvious change in BMD and BV/TV (Fig. 3 *A* and *B*). However, there was no difference in cortical bone (*SI Appendix*, Fig. S3 *C–E* and *H–J*). The H&E staining assay further revealed decreased trabecular bone mass in *REGγ* cKO mice and increased trabecular bone mass in *REGγ* cOE mice compared to their respective control groups (Fig. 3*C* and *SI Appendix*, Fig. S3*K*). The number of TRAP^+^ osteoclasts was significantly increased in bone sections of the *REGγ* cKO mice compared to their *control* littermates and were decreased in *REGγ* cOE mice (Fig. 3 *D* and *E*).

Molecular analysis of bone samples showed increased osteolytic markers, including *Trap*, *Nfatc1*, and *Ctsk,* in *REGγ* cKO mice compared with the controls, while these markers were markedly decreased in *REGγ* cOE mice (Fig. 3*F*). Ex vivo osteoclast differentiation assay revealed an increase osteoclast formation in *REGγ* cKO mice compared to controls, while a decrease formation in *REGγ* cOE mice (*SI Appendix*, Fig. S3 *L* and *M*). These results demonstrate that REGγ regulates osteoclast activity.

### REGγ Suppresses RANKL-Induced Osteoclastogenesis.

**Fig. 4.**
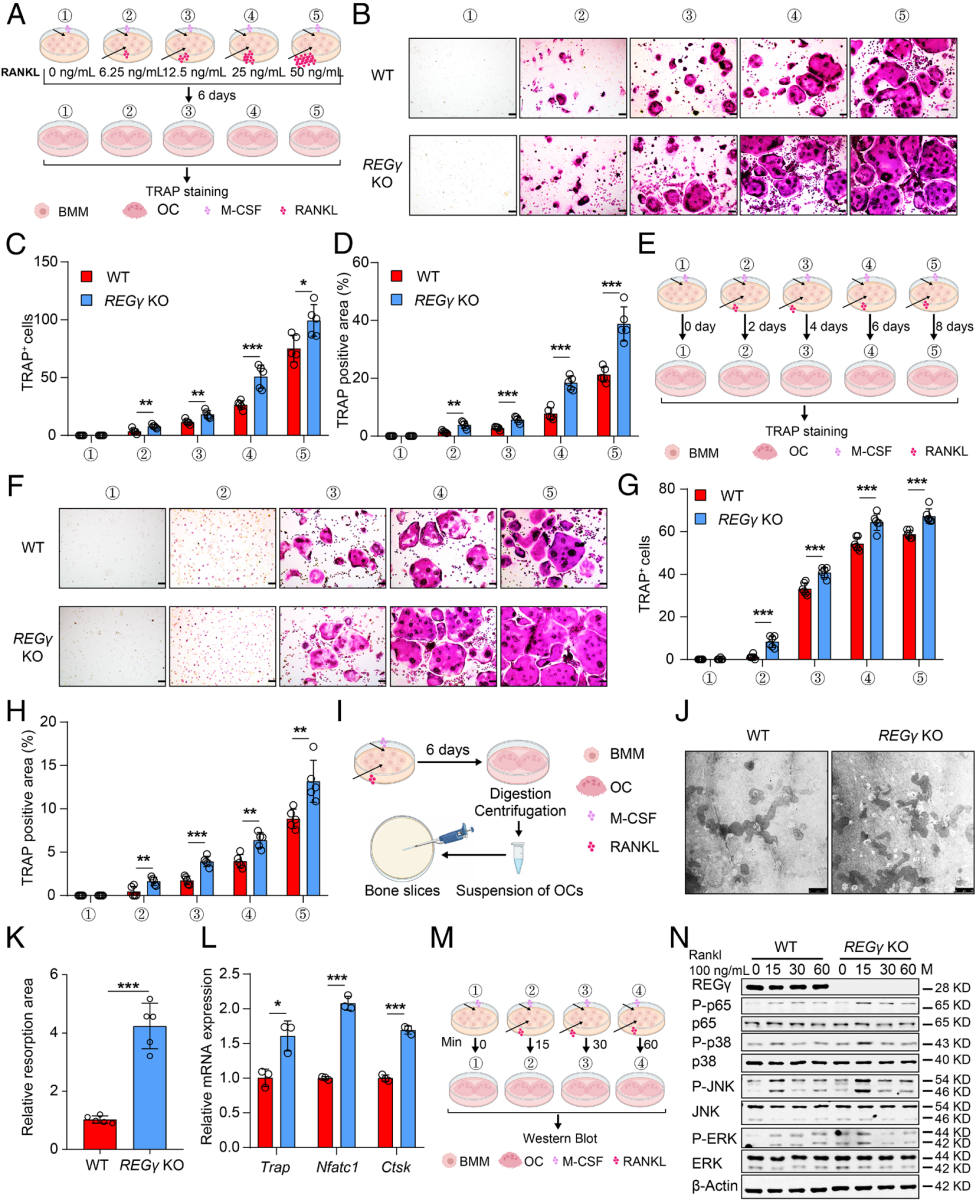
REGγ suppressed RANKL-induced osteoclastogenesis. (*A*) Schematic illustration depicting osteoclast differentiation of BMMs under varying concentrations of RANKL: BMMs were exposed to 10 ng/mL M-CSF along with different concentrations of RANKL to induce osteoclast differentiation over a period of 6 d. The concentrations of RANKL used were 0, 6.25, 12.5, 25, and 50 ng/mL. (*B*) Representative TRAP staining of osteoclasts from WT and *REGγ* KO BMMs treated with RANKL-gradient stimulation. (Scale bar: 100 μm.) (*C* and *D*) Quantification of osteoclast number and TRAP-positive area in (*B*) (n = 5). (*E*) Schematic diagrams illustrating osteoclast differentiation of BMMs at different treatment durations: BMMs were treated with 10 ng/mL M-CSF and 50 ng/mL RANKL to induce osteoclast differentiation over varying time intervals. The time gradients were set to 0, 2, 4, 6, and 8 d. (*F*) Representative TRAP staining of osteoclasts from WT and *REGγ* KO BMMs at various treatment durations. (Scale bar: 100 μm.) (*G* and *H*) Quantification of osteoclast number and TRAP-positive area in (*F*) (n = 5). (*I*) Schematic diagram of pit formation assay: BMMs were treated with 10 ng/mL M-CSF and 50 ng/mL RANKL to induce differentiation for 6 d. Subsequently, mature osteoclasts were harvested and reseeded onto bone slices for a 48-h culture period. (*J*) Representative images of bone erosion pits from WT and *REGγ* KO osteoclasts. (Scale bar: 75 μm.) (*K*) Quantification of the osteoclast resorption area in (*J*) (n = 5). (*L*) Quantification of *Trap*, *Nfatc1*, and *Ctsk* gene expression levels in WT and *REGγ* KO osteoclasts (n = 3). (*M*) Schematic illustration depicting BMM stimulation with RANKL at different times: BMMs were treated with 10 ng/mL M-CSF and 100 ng/mL RANKL to promote activation of the MAPK and NF-κB signaling pathways. The time points were set at 0, 15, 30, and 60 min. (*N*) Western blotting images showing the changes in the MAPK and NF-κB signaling pathways in WT and *REGγ* KO BMMs during RANKL stimulation. This experiment was repeated independently three times. Markers of significance are as follows: N.S, *P* > 0.05; *P < 0.05; **P < 0.01; ***P < 0.001.

RANKL plays a key regulatory role in osteoclast maturation and activation (20–22). To further assess the role of REGγ in osteoclast differentiation, we set up a RANKL concentration gradient for osteoclast induction of BMMs in vitro (Fig. 4*A*). Our results showed that REGγ deficiency promoted the RANKL sensitivity of BMMs (Fig. 4 *B*–*D*). At the same time, we also set a time gradient in vitro for osteoclast induction of BMMs (Fig. 4*E*). REGγ deficiency promoted earlier differentiation of BMMs into osteoclasts (Fig. 4 *F*–*H*). To identify whether REGγ can affect bone resorption, we removed mature osteoclasts to visualize bone resorption pits using Versene (Fig. 4*I*). After 24 h of culture, *REGγ*-null osteoclasts could resorb larger cavities than WT osteoclasts (Fig. 4 *J* and *K*).

Molecular analysis of osteoclasts showed significantly increased osteolytic markers, including *Trap*, *Nfatc1*, and *Ctsk,* in *REGγ* KO mice (Fig. 4*L*). It was reported that RANKL-induced osteoclast differentiation involved activation of the NF-κβ and MAPK pathways downstream of TRAF6 (42–47). We found that the deletion of REGγ significantly increased phosphorylated p65, phosphorylated p38, phosphorylated ERK, and phosphorylated JNK after RANKL stimulation in BMMs (Fig. 4 *M* and *N*).

Therefore, we further analyzed the expression of the aforementioned proteins in the tibia tissues of *REGγ* KO, *REGγ* cKO, and *REGγ* cOE mice (*SI Appendix*, Fig. S4 *A, E*, and *I*). We observed that the levels of phosphorylated p65, phosphorylated p38, phosphorylated ERK, and phosphorylated JNK were elevated in *REGγ*-deficient mice (*SI Appendix*, Fig. S4 *B–D* and *F–H*), whereas the expression of these proteins was reversed in REGγ-overexpressing mice (*SI Appendix*, Fig. S4 *J–L*). Taken together, these findings demonstrated that REGγ can suppress RANKL-induced osteoclastogenesis both in vitro and vivo (*SI Appendix*, Fig. S4*M*).

### REGγ Increases Bone Mass through the Ubiquitin-Independent Degradation of TRAF6.

**Fig. 5.**
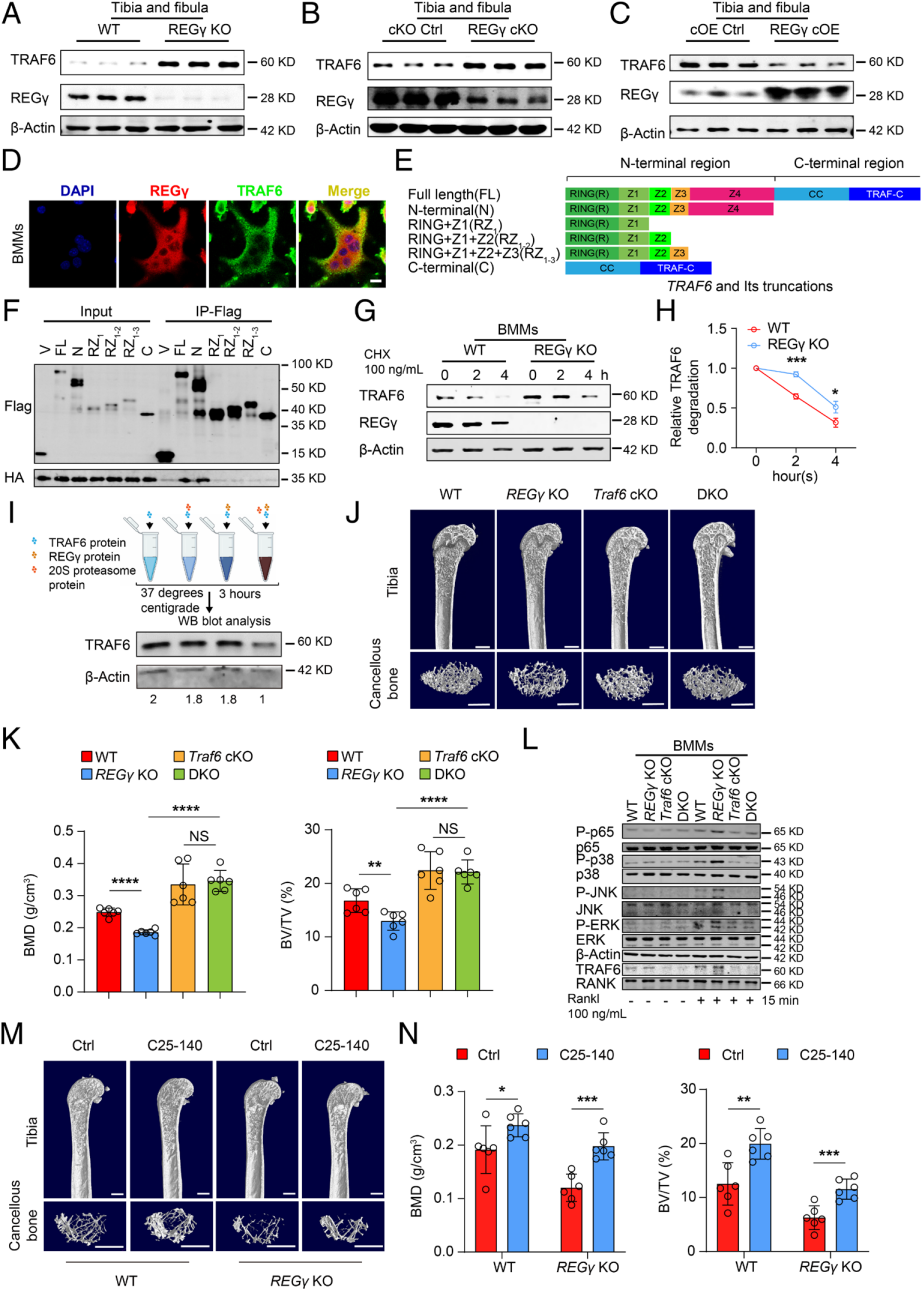
REGγ increased bone mass through ubiquitin-independent degradation of TRAF6. (*A*–*C*) Western blotting analysis of TRAF6 protein levels in WT, *REGγ* KO, cKO Ctrl, *REGγ* cKO, cOE Ctrl, and *REGγ* cOE hindlimb bones. (*D*) Representative images of IF analysis. IF showed overlapping REGγ (red) and TRAF6 (green) in osteoclasts. (Scale bar: 50 μm.) (*E*) Schematic diagram of TRAF6 mutation construction: Domain organization of TRAF6. Z1–Z4: zinc fingers 1 to 4. CC: coiled. FL stands for full-length TRAF6 (residues 1 to 552), RZ_1_ (residues 1 to 159), RZ_12_ (residues 1 to 187), RZ_123_ (residues 1 to 211), N-terminal region (residues 1 to 332), and C-terminal region (residues 333 to 522). (*F*) The interactions between REGγ and TRAF6 were determined by reciprocal coimmunoprecipitation and Western blotting analysis. (*G* and *H*) Western blotting images showing that REGγ can regulate TRAF6 protein stability in BMMs. WT and *REGγ* KO BMMs were treated with cycloheximide (CHX, 100 μg/mL) for the indicated times (*G*), with quantification using ImageJ (*H*) (n = 3). (*I*) Schematic illustration depicting the process of TRAF6 protein degradation in vitro: In vitro proteolytic analyses were performed using purified REGγ, 20S proteasome, and Traf6 protein at 37 °C for 3 h (*Up*). Western blotting images showing that REGγ directly promoted the degradation of TRAF6 in vitro. (*J*) Representative micro-CT images showing the 3D bone structures of femurs from 3-mo-old *REGγ* WT, *REGγ* KO, BMM-conditional *Traf6* KO (*Traf6* cKO), and *Traf6* cKO with *REGγ* KO (DKO) mice. (*K*) Quantification of BMD and BV/TV in femurs by Micro-CT measurement software (n = 6). (Scale bar: 1 mm.) (*L*) Western blotting images depict alterations in TRAF6, RANK, as well as the MAPK and NF-κB signaling pathways in WT, *REGγ* KO, *Traf6* cKO, and DKO BMMs during RANKL stimulation. (*M*) Representative micro-CT images showing the 3D bone structures of femurs from 12-mo-old WT, *REGγ* KO, C25-140-treated WT, and C25-140-treated *REGγ* KO mice. (Scale bar: 1 mm.) (*N*) Micro-CT measurements of BMD and BV/TV in femurs from 12-mo-old WT, *REGγ* KO, C25-140-treated WT, and C25-140-treated *REGγ* KO mice (n = 6). The above experiments were repeated independently three times. Markers of significance are as follows: N.S, *P* > 0.05; *P < 0.05; **P < 0.01; ***P < 0.001. Bone marrow macrophage (BMM)-conditional *Traf6* KO with *REGγ* KO: DKO.

During RANKL-induced osteoclast differentiation, RANKL binds to its receptor RANK, which recruits the adaptor molecule TRAF6, an E3 ubiquitin ligase that regulates downstream NF-kB and MAPK/AP-1 (46). According to the correlation between TRAF6 involved in the ubiquitin-dependent proteasome pathway and REGγ’s function in ubiquitin-independent protein degradation (48–50), we speculate that the positive regulation of REGγ in bone mass is likely through the nonubiquitinated degradation of TRAF6. Therefore, we examined the expression of TRAF6 in the tibia and fibula tissues of *REGγ* KO mice, *REGγ* cKO mice, *REGγ* cOE mice, and their littermates. We observed an upregulation of TRAF6 expression in the tissues of *REGγ* KO mice and *REGγ* cKO mice, while a downregulation was observed in *REGγ* cOE mice (Fig. 5 *A*–*C* and *SI Appendix*, Fig. S5*D*). Similar alterations were detected in the BMMs of mice with above genotypes (*SI Appendix*, Fig. S5 *A* and *B*). In addition, the mRNA levels of *Traf6* were unchanged (*SI Appendix*, Fig. S5*C*). Interestingly, we found that the plasma concentration of TRAF6 was dramatically upregulated in OP patients compared with that in control patients (*SI Appendix*, Fig. S5*E* and Table S2), indicating that the upregulation of TRAF6 could cause osteoporosis. Furthermore, we utilized confocal microscopy to reveal the colocalization of REGγ and TRAF6 in both the nucleus and cytoplasm of osteoclasts (Fig. 5*D*). The nucleoplasmic separation experiments also confirmed this phenomenon (*SI Appendix*, Fig. S5*F*). Then, we generated a series of truncations of *TRAF6* to analyze the REGγ-interactive domain of TRAF6 (Fig. 5*E*). We found that the fourth zinc finger of TRAF6 plays an essential role in the interaction of REGγ and TRAF6 (Fig. 5*F*).

To determine whether REGγ mediates the degradation of TRAF6, we performed a cycloheximide (CHX) chase assay and found that TRAF6 was significantly stabilized in *REGγ* KO BMMs in the presence of CHX (Fig. 5 *G* and *H*). These results further indicated that TRAF6 might be a substrate of REGγ. To verify that REGγ directly degrades TRAF6, we examined the ability of REGγ to direct cell-free proteolysis. Incubation of purified TRAF6 with either the latent 20S proteasome or REGγ alone resulted in no significant degradation of TRAF6 except for nonspecific decay. However, the combination of REGγ and the 20S proteasome promoted significant degradation of TRAF6 (Fig. 5*I*). Taken together, these data revealed that REGγ could regulate the degradation of TRAF6 in a ubiquitin-independent manner.

To ascertain the TRAF6 dependency of the REGγ-osteoclast phenomenon, we generated double KO (DKO) mice by crossing bone marrow-derive macrophage (BMM)-conditional *Traf6* KO mice with *REGγ* KO mice (*SI Appendix*, Fig. S5*G*). We found that DKO mice exhibited higher bone mass compared to *REGγ* KO mice (Fig. 5*J*). This observation was further confirmed by H&E staining. In addition, the TRAP staining results indicated a decreased TRAP-positive rate in the femur sections of DKO mice (*SI Appendix*, Fig. S5 *H* and *I*). Ex vivo osteoclast differentiation assay revealed a decrease osteoclast formation in DKO mice compared to *REGγ* KO mice (*SI Appendix*, Fig. S5 *J* and *K*).

Furthermore, we also found that the double deletion of *REGγ* and *Traf6* significantly decreased phosphorylated p65, phosphorylated p38, phosphorylated ERK, and phosphorylated JNK after RANKL stimulation in BMMs compared with deletion of *REGγ* (Fig. 5*L* and *SI Appendix*, Fig. S5*L*). The same results were obtained in bone tissue (*SI Appendix*, Fig. S6 *A* and *B*). Collectively, these data indicated that TRAF6 deletion rescued the enhanced osteoclast activity and reduced bone mass caused by REGγ deficiency, suggesting that the REGγ-osteoclast phenomenon was TRAF6-dependent. However, another REGγ substrate, p21, had minimal impact on this phenomenon (*SI Appendix*, Fig. S6 *C–F*).

C25-140 is a TRAF6 inhibitor that disrupts the interaction between TRAF6 and Ubc13, directly affecting the activity of TRAF6 (51). To investigate its potential in treating osteoporosis, we administered C25-140 treatment and observed its inhibitory effect on osteoclast differentiation in both WT and *REGγ* KO BMMs (*SI Appendix*, Fig. S6 *G–J*). Considering that in vivo experiments are more illustrative, we administered C25-140 to *REGγ* KO mice via intraperitoneal injection for 1 mo (*SI Appendix*, Fig. S6*K*). We found that bone mass was higher in the treated mice than in the saline-injected group with no weight change (Fig. 5 *M* and *N* and *SI Appendix*, Fig. S6*L*), and the number of osteoclasts in the treated mice was lower (*SI Appendix*, Fig. S6 *M* and *N*).

In summary, REGγ degrades TRAF6 to maintain physiological homeostasis. In the absence of REGγ, the expression level of TRAF6 increases, triggering the activation of osteoclast differentiation pathways and subsequent bone resorption. The deficiency of TRAF6 rescues this phenomenon. Similarly, the TRAF6 inhibitor C25-140 exhibits analogous effects, suggesting its potential as a unique therapeutic strategy for treating osteoporosis (*SI Appendix*, Fig. S6*O*).

### NIP30 Dephosphorylation Activates the Ubiquitin-Independent Degradation of TRAF6 to Alleviate Osteoporosis.

**Fig. 6.**
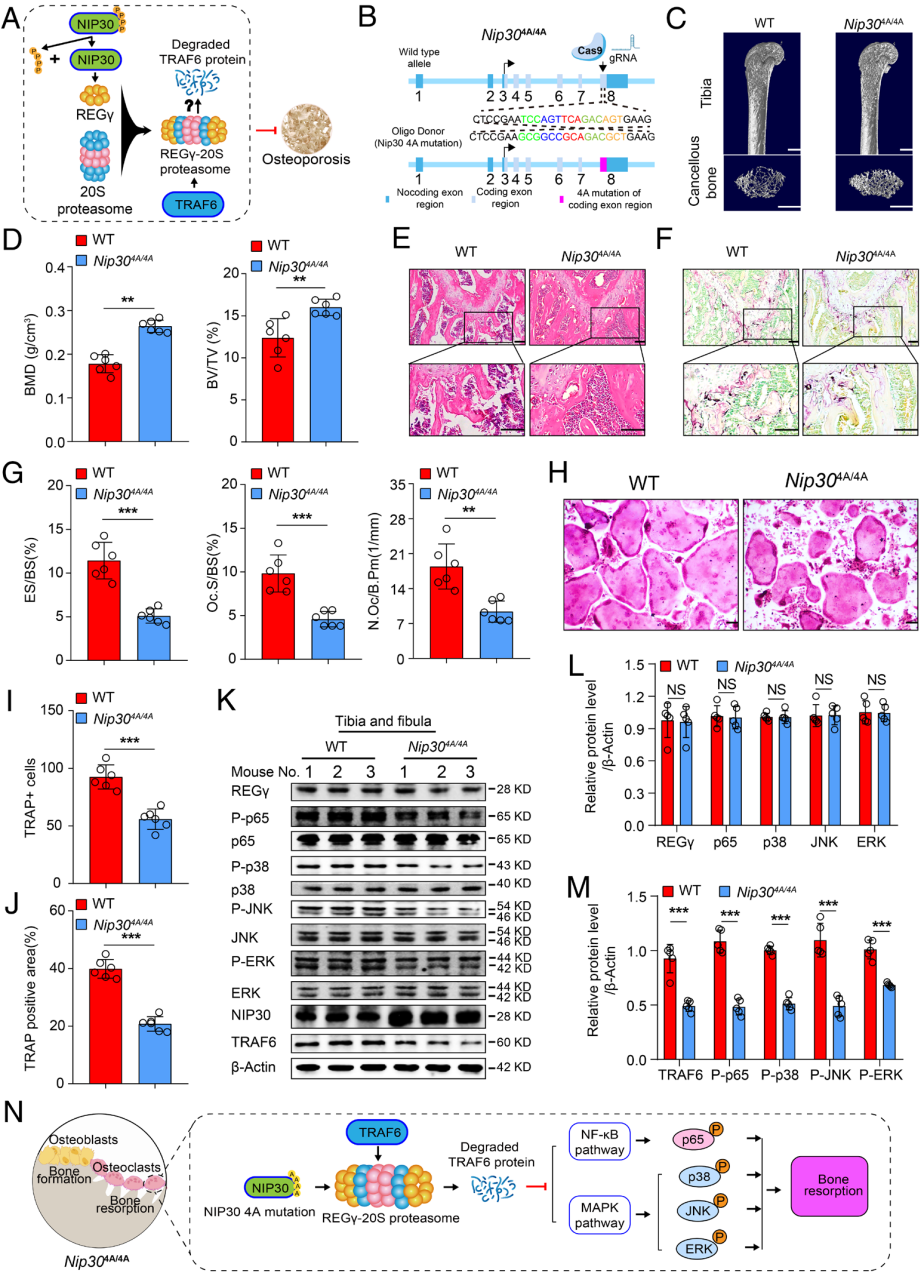
NIP30 dephosphorylation activates the ubiquitin-independent degradation of TRAF6 to alleviate osteoporosis. (*A*) Hypothetical schematic diagram illustrating the involvement of NIP30 in the regulation of osteoporosis: NIP30 acts as an upstream regulator of REGγ. When serine residues at positions 226 to 230 of NIP30 are dephosphorylation, it fails to bind to REGγ and promote the function of the REGγ-20S proteasome. This alteration may impact the ubiquitin-independent degradation of TRAF6, thereby regulating the development of osteoporosis. (*B*) Strategy diagram illustrating the generational design of *Nip30*^4A/4A^ mice. (*C*) Representative micro-CT images showing the 3D bone structures of femurs from 2-mo-old WT and *Nip30*^A/4A^ mice (n = 6). (Scale bar: 1 mm.) (*D*) Micro-CT measurements of BMD and BV/TV in femurs from 2-mo-old WT and *Nip30*^4A/4A^ mice (n = 6). (*E*) Representative images of H&E staining of femurs of 2-mo-old WT and *Nip30*^4A/4A^ mice (n = 6). (Scale bar: 100 μm.) (*F*) Representative immunofluorescence images of TRAP expression in 2-mo-old WT and *Nip30*^4A/4A^ mice (n = 6). (Scale bar: 100 μm.) (*G*) Histomorphometrical analysis of TRAP staining in (*F*) (n = 6). (*H*) Representative TRAP staining of osteoclasts from WT and *Nip30*^4A/4A^ BMMs treated with RANKL stimulation. (Scale bar: 100 μm.) (*I* and *J*) Quantification of osteoclast number and TRAP-positive area in (*H*) (n = 5). (*K*–*M*) Western blotting images depict alterations in REGγ, TRAF6, NIP30, as well as the MAPK and NF-κB signaling pathways in the hindlimb bones of WT and *Nip30*^4A/4A^ mice (*K*). The above results were analyzed using ImageJ (*L* and *M*) (n = 5). (*N*) Schematic diagram illustrating the mechanism of bone resorption in *Nip30*^4A/4A^ mice: The NIP30 4A mutation inhibits the function of the REGγ-20S proteasome, thereby promoting ubiquitin-independent degradation of TRAF6, leading to inhibition of the MAPK and NF-κB signaling pathways, and consequently suppressing the occurrence of osteoporosis. Markers of significance are as follows: N.S, *P* > 0.05; *P < 0.05; **P < 0.01; ***P < 0.001.

Considering that REGγ is a positive regulator of bone mass, we tried to find molecules that could augment the function of the REGγ-20S proteasome. Our previous work found that NIP30 cannot bind to REGγ to promote the function of REGγ-20S proteasome after mutating four serine lines at positions 226-230 to alanine, suggesting that inhibiting the phosphorylation of NIP30 is an "activator" of the REGγ-20S proteasome (35, 36). It may be possible to upregulate bone mass by inhibiting NIP30 phosphorylation (Fig. 6*A*). To investigate whether the NIP30/REGγ pathway regulates osteoporosis, we first constructed *Nip30* 4A (mimic loss function of NIP30) transgenic mice (Fig. 6*B* and *SI Appendix*, Fig. S7*A*). We found that the BMD and bone volume of *Nip30*^4A/4A^ mice showed an increase in cancellous bone compared to that of WT mice (Fig. 6 *C* and *D*), but there was little change in cortical bone (*SI Appendix*, Fig. S7 *B* and *C*). The same phenomenon was demonstrated by H&E staining (Fig. 6*E* and *SI Appendix*, Fig. S7*D*). Moreover, the number of osteoclasts and osteoclastic markers in the bones of *Nip30*^4A/4A^ mice appeared to be significantly lower compared to those in WT mice (Fig. 6 *F* and *G*) and revealed a decrease osteoclast differentiation of *Nip30*^4A/4A^ BMMs compared to that of WT mice (Fig. 6 *H*–*J* and *SI Appendix*, Fig. S7 *E* and *F*). Meanwhile, there is no difference of bone formation in vitro and in vivo (*SI Appendix*, Fig. S7 *G–I*).

Next, we examined TRAF6 expression in bone tissues and BMMs of both *Nip30*^4A/4A^ and WT mice. We observed a downregulation of TRAF6 expression in *Nip30*^4A/4A^ mice (*SI Appendix*, Fig. S7 *J* and *K*), suggesting that NIP30/REGγ/TRAF6 pathway regulated osteoporosis. Furthermore, we also found that phosphorylation of p65, p38, ERK, and JNK was reduced in the bone tissue of *Nip30*^4A/4A^ mice compared to WT mice (Fig. 6 *K*–*M*).

To ascertain the REGγ dependency of the Nip30 4A-osteoclast phenomenon, we generated *REGγ* KO *Nip30*^4A/4A^ mice by crossing *Nip30*^4A/4A^ mice with *REGγ* KO mice. Ex vivo osteoclast differentiation assay revealed an increase osteoclast formation of *REGγ* KO *Nip30*^4A/4A^ mice compared to that of *Nip30*^4A/4A^ mice (*SI Appendix*, Fig. S7 *L–N*). Additionally, we found that compared to *Nip30*^4A/4A^ mice, *REGγ* KO *Nip30*^4A/4A^ mice exhibited increased expression of TRAF6 in bone tissue, accompanied by elevated phosphorylation levels of p65, p38, ERK, and JNK (*SI Appendix*, Fig. S7 *O–Q*). Taken together, these results suggested that NIP30 dephosphorylation inhibits the function of the REGγ-20S proteasome, thereby promoting ubiquitin-independent degradation of TRAF6 to inhibit the development of osteoporosis (Fig. 6*N*).

### TTP22 Alleviates Osteoporosis by Regulating the CKII/NIP30/REGγ/TRAF6 Axis.

**Fig. 7.**
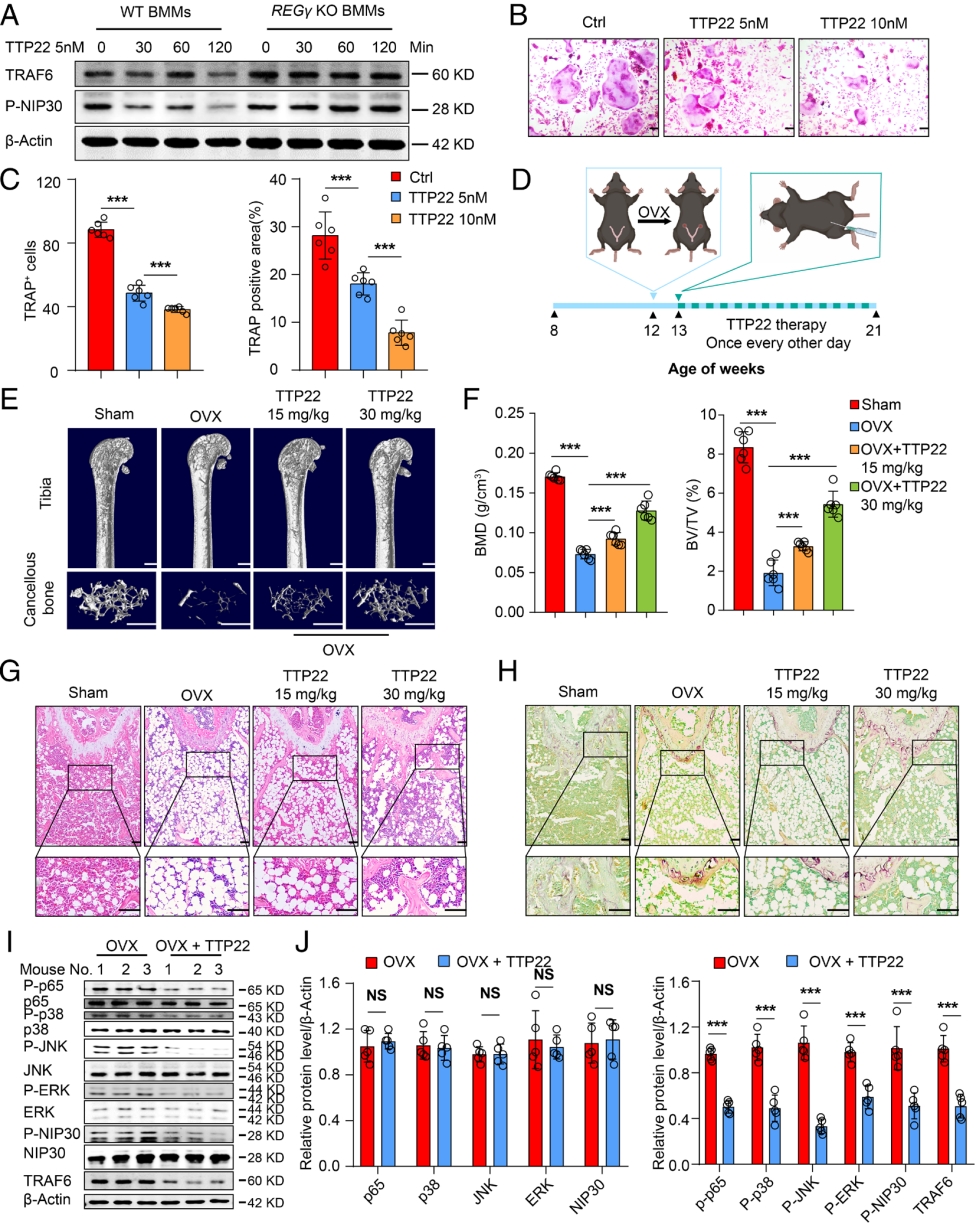
TTP22 alleviates osteoporosis by regulating the CKII/NIP30/REGγ/TRAF6 axis. (*A*) Western blotting analysis of the protein levels of P-NIP30 and TRAF6 with or without TTP22 treatment (0, 30, 60, 120 min). (*B*) Representative TRAP staining of BMMs from WT mice treated with or without TTP22. (Scale bar: 100 μm.) (*C*) Quantification of osteoclast number and TRAP-positive area in (*B*). (*D*) Schematic diagram illustrating the experimental design of TTP22 treatment in WT mice: One week after OVX surgery, mice were subjected to intraperitoneal injections of TTP22 every other day for a total of 8 wk. (*E*) Representative micro-CT images showing the 3D bone structures of femurs from 5-mo-old Sham, OVX, and TTP22-treated WT mice (n = 6). (Scale bar: 1 mm.) (*F*) Micro-CT measurements of BMD and BV/TV in femurs from 5-mo-old Sham, OVX, and TTP22-treated WT mice (n = 6). (*G*) Representative images of H&E staining of femurs of 5-mo-old Sham, OVX, and TTP22-treated WT mice (n = 6). (Scale bar: 100 μm.) (*H*) Representative immunofluorescence images of TRAP expression in 5-mo-old Sham, OVX, and TTP22-treated WT mice (n = 6). (Scale bar: 100 μm.) (*I* and *J*) Western blotting images depict alterations in TRAF6, NIP30, and P-NIP30 as well as the MAPK and NF-κB signaling pathways in the hindlimb bones of OVX and TTP22-treated WT mice (*I*). The above results were analyzed using ImageJ (*J*) (n = 5). Markers of significance are as follows: N.S, *P* > 0.05; *P < 0.05; **P < 0.01; ***P < 0.001.

Several studies have shown that CKII is an upstream kinase of NIP30 and can promote NIP30 phosphorylation (35, 36). Considering that the NIP30/REGγ/TRAF6 axis is critical in osteoporosis, we used TTP22 to determine whether it could inhibit the phosphorylation of NIP30 to affect the function of REGγ (*SI Appendix*, Fig. S7*R*). Interestingly, we found that TTP22 inhibited the phosphorylation of NIP30 and attenuated the expression of TRAF6 in BMMs of WT mice (Fig. 7*A*). In addition, we found that another inhibitor of CKII, CX4945, could also inhibit the phosphorylation of NIP30 and attenuate the expression of TRAF6 in BMMs of WT and *REGγ* KO mice (*SI Appendix*, Fig. S8*A*). However, the function of CX4945 was not REGγ dependent. Subsequently, TTP22 intervention was performed during osteoclast differentiation, and TTP22 was found to inhibit the ability of BMMs to differentiate toward osteoclasts (Fig. 7 *B* and *C* and *SI Appendix*, Fig. S8*H*). These results may imply that TTP22 is able to regulate bone mass by affecting osteoclast differentiation.

To determine the in vivo therapeutic potential of TTP22 in bone resorption-related diseases, TTP22 was intraperitoneally injected every other day in OVX mice (Fig. 7*D*). Subsequently, we analyzed the drug metabolism by pharmacokinetics and found that the drug concentration in blood was still at an effective concentration 24 h after injection, indicating that TTP22 has good potential for application (*SI Appendix*, Fig. S8*B*). Two months after the first injection, the body weights of the mice were barely affected (*SI Appendix*, Fig. S8*C*). H&E staining of internal tissues also showed no significant changes in tissue morphology (*SI Appendix*, Fig. S8*D*), indicating that TTP22 had no significant side effects. Interestingly, micro-CT analysis showed that the bone mass of TTP22-treated OVX mice significantly increased compared to that of untreated OVX mice (Fig. 7*E*). The quantitative values of the BV/TV and BMD significantly increased in the femurs of TTP22-treated mice (Fig. 7*F*). However, there was no difference in cortical bone (*SI Appendix*, Fig. S8 *E* and *F*). Bone histomorphometric analysis with TRAP staining showed that the number of osteoclasts in OVX mice decreased after TTP22 treatment (Fig. 7 *G* and *H*). Additionally, compared to OVX mice, OVX mice treated with TTP22 exhibited a noticeable reduction in osteoclastic markers in their bones (*SI Appendix*, Fig. S8*G*). Meanwhile, there were no differences in bone formation observed both in vitro and in vivo (*SI Appendix*, Fig. S8 *I–K*).

Furthermore, Western blot results also demonstrated a decrease in TRAF6 and P-NIP30 expression after TTP22 treatment, accompanied by a reduction in the phosphorylation levels of proteins associated with the MAPK and NF-κB signaling pathways (Fig. 7 *I* and *J*). Taken together, our data demonstrate that TTP22 can alleviate osteoporosis by inhibiting NIP30/REGγ/TRAF6.

## Discussion

The proteasome system plays essential roles in numerous cellular processes, and its dysfunction may result in many diseases, such as multiple myeloma and autoimmune diseases, making it a considerable therapeutic target (52–54). The proteasome system can be divided into two groups: the ubiquitin-dependent 19S proteasome activators and the ubiquitin-independent 11S proteasome family (PSME1, PSME2, and PSME3, also named REGγ), the latter of which is a set of conserved proteins that promote ATP-independent protein degradation (55, 56). Previous studies have given much attention to the ubiquitin–proteasome system, but the ubiquitin-independent proteasome system has received increasing attention in recent years. In this study, we investigated the role of REGγ in osteoporosis in detail, demonstrating that REGγ could be a potential predictive biomarker for the early diagnosis and modulation of REGγ activity by interfering with NIP30-REGγ interactions, which may provide unique venues for the development of pharmacological agents.

To the best of our knowledge, in addition to our previous finding that REGγ deficiency manifests as premature aging, including osteoporosis (34), few studies have explored the regulatory mechanism between the ubiquitin-independent proteasome system and osteoporosis. In this study, we revealed that the expression of REGγ is negatively related to osteoporosis and that REGγ deficiency aggravates osteoporosis by promoting the activation of osteoclasts. Interestingly, we found the expression of REGγ gradually decreases in osteoclast differentiation process, thereby exacerbating osteoporosis. Based on our results, we believe that REGγ could be a biomarker predicting individual susceptibility to osteoporosis and provide a different venue for treating osteoporosis by interfering with the REGγ-20S proteasome.

Our team provided the evidence that the REGγ-20S proteasome can degrade intact cellular proteins (57). To date, multiple important regulatory proteins have been found to degrade in this ubiquitin-independent manner, such as p21, p16, p19, and p53 (31, 56–58). However, the downstream targets of the REGγ-20S proteasome involved in the progression of osteoporosis and osteoclastogenesis remain unclear. One of the most striking features of the present study is that we identified TRAF6 as a pivotal downstream target of the REGγ-20S proteasome involved in regulating osteoclast activation in the context of osteoporosis. TRAF6 is essential for many biological processes, including the activation of osteoclasts (59). As a central upstream adaptor, TRAF6 regulates osteoclastogenesis by orchestrating RANKL/RANK and downstream signaling, such as the NF-κβ, MAPKs, and PI3K axes (23, 25, 27, 42, 60–62). Although there is evidence that the degradation of TRAF6 requires the ubiquitin-dependent proteasome system (63), another study suggested that TRAF6 could be selectively degraded via autophagy (64). Our results further illustrated that the REGγ-20S proteasome directly degrades the E3 ligase TRAF6, indicating that the alternative ubiquitin-independent proteasome pathway is an extremely attractive protein degradation mechanism and that the regulation of the REGγ-20S proteasome/TRAF6 pathway could be a potential strategy for osteoporosis treatment.

NIP30, recently identified as a REGγ “inhibitor,” functions to modulate REGγ in cells (35, 36). The highly specific binding of NIP30 to REGγ inhibits ubiquitin-independent proteasome-degrading proteins and depends on the phosphorylation of NIP30. There are no effective molecular agents regulating the function of NIP30 or REGγ. Our previous study showed that CKII could phosphorylate the C-terminus of NIP30 (36), indicating that fine-tuning the NIP30/REGγ/TRAF6 axis is a potential strategy to treat osteoporosis. In our study, we utilized TTP22, a selective inhibitor of CKII, to test the hypothesis that it could suppress osteoclastogenesis and treat osteoporosis. The results showed that TTP22 effectively restrained the progression of osteoporosis in the OVX mouse model with favorable biological safety, indicating potential for its clinical application in the treatment of osteoporosis.

In conclusion, our study reveals REGγ as a therapeutic biomarker of osteoporosis. Based on the pivotal mechanism of the NIP30/REGγ/TRAF6 axis, we determined that TTP22 can alleviate osteoporosis by promoting the ubiquitin-independent proteasomal degradation of TRAF6 and provide a different strategy for the treatment of osteoporosis.

## Materials and Methods

### Human Samples.

Sample collections were performed after written informed consent. To protect patient privacy, all personal information was de-identified. Institutional approval was obtained from the Shanghai General Hospital Institutional Review Board (Approval No. 2021094). The standards for sample collection are presented in *SI Appendix*.

### Animals.

*REGγ* KO mice with a C57BL/6 genetic background were a friendly gift from Dr. John J. Monaco of the University of Cincinnati, and have been backcrossed in our facility for over 10 generations. *REGγ*^fl/fl^, LSL-*REGγ*, and *LysM*-Cre mice were generated as described elsewhere (1, 2). To generate *LysM*-Cre/*Traf6*^fl/fl^/*REGγ* KO (DKO) mice, *REGγ* KO mice were first crossed with *Traf6*^fl/fl^ mice and *LysM*-Cre mice separately to generate *Traf6*^fl/fl^/*REGγ* KO mice and *LysM*-Cre/*REGγ* KO mice. Subsequently, these mice were further crossed together to bring out *LysM*-Cre/*Traf6*^fl/fl^/*REGγ* KO genotype. *Nip30*^4A/4A^ mouse line was used in the current study. *Nip30*^4A/4A^ mice were designed and constructed in our laboratory. *REGγ* KO *Nip30*^4A/4A^ mice were generated by crossing *Nip30*^4A/4A^ mice with *REGγ* KO mice. All mice were housed in a specific pathogen-free (SPF) facility following standard humane animal husbandry protocols, approved by the animal care and use committee of the Institute of Microbiology (Chinese Academy of Sciences).

Human samples information, Plasmids information, micro-CT assays, immunohistochemistry assays, immunofluorescence assays, In vitro osteoclast differentiation assays, In vitro osteoblast differentiation assay, TRAP staining assay, pit formation assay, ALP staining, Alizarin Red S staining and Von Kossa staining, RNA extraction and Real-Time Quantitative PCR assays, western blotting assay, Co-immunoprecipitation assay, in vitro proteolytic analysis assay, and Enzyme-Linked Immunosorbent Assay, as well as reagents and resources, are listed in *SI Appendix*, *Materials and Methods*.

## Supplementary Material

Appendix 01 (PDF)

## Data Availability

The mass spectrometry data is being deposited to the ProteomeXchange Consortium via the iProX repository with the data identifier IPX0010088000 (ProteomeXchange dataset Identifier: PXD057237) (65). All other data are included in the article and/or *SI Appendix*.

## References

[1] P. N. Sambrook, Osteoporosis. Med. J. Aust. **165**, 332–336 (1996). 8862335

[2] B. Seriolo , Osteoporosis in the elderly. Aging Clin. Exp. Res. **1**, S27–S29 (2013). 10.1007/s40520-013-0107-9 23963883

[3] I. R. Reid, A broader strategy for osteoporosis interventions. Nat. Rev. Endocrinol. **16**, 333–339 (2020). 32203407 10.1038/s41574-020-0339-7

[4] E. Lau, H. Chung, P. Ha, H. Tang, D. Lam, Bone mineral density, anthropometric indices, and the prevalence of osteoporosis in Northern (Beijing) Chinese and Southern (Hong Kong) Chinese women–The largest comparative study to date. J. Clin. Densitom. **18**, 519–524 (2015). 25592395 10.1016/j.jocd.2014.11.001

[5] P. Xiao , Global, regional prevalence, and risk factors of osteoporosis according to the World Health Organization diagnostic criteria: A systematic review and meta-analysis. Osteoporos. Int. **33**, 2137–2153 (2022). 35687123 10.1007/s00198-022-06454-3

[6] J. Kanis, Diagnosis of osteoporosis and assessment of fracture risk. Lancet **359**, 1929–1936 (2002). 12057569 10.1016/S0140-6736(02)08761-5

[7] J. Compston, M. McClung, W. Leslie, Osteoporosis. Lancet **393**, 364–376 (2019). 30696576 10.1016/S0140-6736(18)32112-3

[8] S. Sattui, K. Saag, Fracture mortality: Associations with epidemiology and osteoporosis treatment. Nat. Rev. Endocrinol. **10**, 592–602 (2014). 25091729 10.1038/nrendo.2014.125

[9] T. Vilaca, R. Eastell, M. Schini, Osteoporosis in men. Lancet Diabetes Endocrinol. **10**, 273–283 (2022). 35247315 10.1016/S2213-8587(22)00012-2

[10] T. Kuo, C. Chen, Bone biomarker for the clinical assessment of osteoporosis: Recent developments and future perspectives. Biomark. Res. **5**, 18 (2017). 28529755 10.1186/s40364-017-0097-4 PMC5436437

[11] F. Migliorini , Biomarkers as therapy monitoring for postmenopausal osteoporosis: A systematic review. J. Orthop. Surg. Res. **16**, 318 (2021). 34006294 10.1186/s13018-021-02474-7 PMC8130375

[12] D. König, S. Oesser, S. Scharla, D. Zdzieblik, A. Gollhofer, Specific collagen peptides improve bone mineral density and bone markers in postmenopausal women-a randomized controlled study. Nutrients **10**, 97 (2018). 29337906 10.3390/nu10010097 PMC5793325

[13] M. McClung , Romosozumab in postmenopausal women with low bone mineral density. N. Engl. J. Med. **370**, 412–420 (2014). 24382002 10.1056/NEJMoa1305224

[14] I. Reid, E. Billington, Drug therapy for osteoporosis in older adults. Lancet **399**, 1080–1092 (2022). 35279261 10.1016/S0140-6736(21)02646-5

[15] S. Khosla, L. Hofbauer, Osteoporosis treatment: Recent developments and ongoing challenges. Lancet Diabetes Endocrinol. **5**, 898–907 (2017). 28689769 10.1016/S2213-8587(17)30188-2 PMC5798872

[16] W. Deardorff, I. Cenzer, B. Nguyen, S. Lee, Time to benefit of bisphosphonate therapy for the prevention of fractures among postmenopausal women with osteoporosis: A meta-analysis of randomized clinical trials. JAMA Intern. Med. **182**, 33–41 (2022). 34807231 10.1001/jamainternmed.2021.6745 PMC8609461

[17] S. Song, Y. Guo, Y. Yang, D. Fu, Advances in pathogenesis and therapeutic strategies for osteoporosis. Pharmacol. Ther. **237**, 108168 (2022). 35283172 10.1016/j.pharmthera.2022.108168

[18] Y. Ikebuchi , Coupling of bone resorption and formation by RANKL reverse signalling. Nature **561**, 195–200 (2018). 30185903 10.1038/s41586-018-0482-7

[19] L. Wang, X. You, L. Zhang, C. Zhang, W. Zou, Mechanical regulation of bone remodeling. Bone Res. **10**, 16 (2022). 35181672 10.1038/s41413-022-00190-4 PMC8857305

[20] S. Khosla, Minireview: The OPG/RANKL/RANK system. Endocrinology **142**, 5050–5055 (2001). 11713196 10.1210/endo.142.12.8536

[21] S. Teitelbaum , Genetic regulation of osteoclast development and function. Nat. Rev. Genet. **4**, 638–649 (2003). 12897775 10.1038/nrg1122

[22] N. Takegahara , RANKL biology. Bone **159**, 116353 (2022). 35181574 10.1016/j.bone.2022.116353 PMC9035122

[23] S. Strickson , Roles of the TRAF6 and Pellino E3 ligases in MyD88 and RANKL signaling. Proc. Natl. Acad. Sci. U.S.A. **114**, E3481–E3489 (2017). 28404732 10.1073/pnas.1702367114 PMC5410814

[24] K. Yoon , TRAF6 deficiency promotes TNF-induced cell death through inactivation of GSK3beta. Cell Death Differ. **15**, 730–738 (2008). 18202703 10.1038/sj.cdd.4402304

[25] H. Takayanagi , T-cell-mediated regulation of osteoclastogenesis by signalling cross-talk between RANKL and IFN-gamma. Nature **408**, 600–605 (2000). 11117749 10.1038/35046102

[26] P. Lai , Loss of Rictor with aging in osteoblasts promotes age-related bone loss. Cell Death Dis. **7**, e2408 (2016). 27735936 10.1038/cddis.2016.249 PMC5133960

[27] M. Lomaga , TRAF6 deficiency results in osteopetrosis and defective interleukin-1, CD40, and LPS signaling. Genes Dev. **13**, 1015–1024 (1999). 10215628 10.1101/gad.13.8.1015 PMC316636

[28] V. Deepak , IFT80 negatively regulates osteoclast differentiation via association with Cbl-b to disrupt TRAF6 stabilization and activation. Proc. Natl. Acad. Sci. U.S.A. **119**, e2201490119 (2022). 35733270 10.1073/pnas.2201490119 PMC9245634

[29] H. Jang, H. Hwang, H. Kim, S. Lee, C-Cbl negatively regulates TRAF6-mediated NF-κB activation by promoting K48-linked polyubiquitination of TRAF6. Cell Mol. Biol. Lett. **24**, 29 (2019). 31123462 10.1186/s11658-019-0156-y PMC6518801

[30] T. Nikaido , Cloning and nucleotide sequence of cDNA for Ki antigen, a highly conserved nuclear protein detected with sera from patients with systemic lupus erythematosus. Clin. Exp. Immunol. **79**, 209–214 (1990). 1968796 10.1111/j.1365-2249.1990.tb05180.x PMC1534747

[31] I. Mao, J. Liu, X. Li, H. Luo, REGgamma, a proteasome activator and beyond? Cell Mol. Life Sci. **65**, 3971–3980 (2008). 18679578 10.1007/s00018-008-8291-z PMC11131756

[32] L. Barton , Immune defects in 28-kDa proteasome activator gamma-deficient mice. J. Immunol. Res. **172**, 3948–3954 (2004). 10.4049/jimmunol.172.6.3948 15004203

[33] S. Murata , Growth retardation in mice lacking the proteasome activator PA28gamma. J. Biol. Chem. **274**, 38211–38215 (1999). 10608895 10.1074/jbc.274.53.38211

[34] L. Li , REGγ deficiency promotes premature aging via the casein kinase 1 pathway. Proc. Natl. Acad. Sci. U.S.A. **110**, 11005–11010 (2013). 23766372 10.1073/pnas.1308497110 PMC3703992

[35] B. Jonik-Nowak , PIP30/FAM192A is a novel regulator of the nuclear proteasome activator PA28γ. Proc. Natl. Acad. Sci. U.S.A. **115**, E6477–E6486 (2018). 29934401 10.1073/pnas.1722299115 PMC6048556

[36] X. Gao , The REGγ inhibitor NIP30 increases sensitivity to chemotherapy in p53-deficient tumor cells. Nat. Commun. **11**, 4888 (2020). 32968062 10.1038/s41467-020-18767-0 PMC7511305

[37] Q. Ma , Osteoclast-derived apoptotic bodies couple bone resorption and formation in bone remodeling. Bone Res. **9**, 5 (2021). 33431863 10.1038/s41413-020-00121-1 PMC7801485

[38] C. Li , The osteoprotective role of USP26 in coordinating bone formation and resorption. Cell Death Differ. **29**, 1123–1136 (2022). 35091692 10.1038/s41418-021-00904-x PMC9177963

[39] J. Delgado-Calle, T. Bellido, The osteocyte as a signaling cell. Physiol. Rev. **102**, 379–410 (2022). 34337974 10.1152/physrev.00043.2020 PMC8858675

[40] X. Zhu , REGγ drives Lgr5+ stem cells to potentiate radiation induced intestinal regeneration. Sci. China Life Sci. **65**, 1608–1623 (2022). 34826093 10.1007/s11427-021-2018-7

[41] J. Tu , Aging-associated REGγ proteasome decline predisposes to tauopathy. J. Biol. Chem. **298**, 102571 (2022). 36209822 10.1016/j.jbc.2022.102571 PMC9647549

[42] N. Kobayashi , Segregation of TRAF6-mediated signaling pathways clarifies its role in osteoclastogenesis. EMBO J. **20**, 1271–1280 (2001). 11250893 10.1093/emboj/20.6.1271 PMC145527

[43] G. Franzoso , Requirement for NF-kappaB in osteoclast and B-cell development. Genes Dev. **11**, 3482–3496 (1997). 9407039 10.1101/gad.11.24.3482 PMC316809

[44] Y. Chen , Mogrol attenuates osteoclast formation and bone resorption by inhibiting the TRAF6/MAPK/NF-κB signaling pathway in vitro and protects against osteoporosis in postmenopausal mice. Front. Pharmacol. **13**, 803880 (2022). 35496311 10.3389/fphar.2022.803880 PMC9038946

[45] K. Sun , Gamabufotalin inhibits osteoclastgenesis and counteracts estrogen-deficient bone loss in mice by suppressing RANKL-induced NF-κB and ERK/MAPK pathways. Front. Pharmacol. **12**, 629968 (2021). 33967763 10.3389/fphar.2021.629968 PMC8104077

[46] E. Tan, L. Li, I. Indran, N. Chew, E. Yong, TRAF6 mediates suppression of osteoclastogenesis and prevention of ovariectomy-induced bone loss by a novel prenylflavonoid. J. Bone Miner. Res. **32**, 846–860 (2017). 27813153 10.1002/jbmr.3031

[47] K. Lee , Selective regulation of MAPK signaling mediates RANKL-dependent osteoclast differentiation. Int. J. Biol. Sci. **12**, 235–245 (2016). 26884720 10.7150/ijbs.13814 PMC4737679

[48] V. Deepak , IFT80 negatively regulates osteoclast differentiation via association with Cbl-b to disrupt TRAF6 stabilization and activation. Proc. Natl. Acad. Sci. U.S.A. **119**, e2201490119 (2022). 35733270 10.1073/pnas.2201490119 PMC9245634

[49] L. Tong , Proteasome-dependent degradation of Smad7 is critical for lung cancer metastasis. Cell Death Differ. **27**, 1795–1806 (2020). 31767934 10.1038/s41418-019-0459-6 PMC7244558

[50] L. Li , REGγ is critical for skin carcinogenesis by modulating the Wnt/β-catenin pathway. Nat. Commun. **6**, 6875 (2015). 25908095 10.1038/ncomms7875

[51] J. Brenke , Targeting TRAF6 E3 ligase activity with a small-molecule inhibitor combats autoimmunity. J. Biol. Chem. **293**, 13191–13203 (2018). 29950522 10.1074/jbc.RA118.002649 PMC6109917

[52] L. Fricker, Proteasome inhibitor drugs. Annu. Rev. Pharmacol. Toxicol. **60**, 457–476 (2020). 31479618 10.1146/annurev-pharmtox-010919-023603

[53] T. Thibaudeau, D. Smith, A practical review of proteasome pharmacology. Pharmacol. Rev. **71**, 170–197 (2019). 30867233 10.1124/pr.117.015370 PMC6423620

[54] J. Wang, Y. Fang, R. Fan, C. Kirk, Proteasome inhibitors and their pharmacokinetics, pharmacodynamics, and metabolism. Int. J. Mol. Sci. **22**, 11595 (2021). 34769030 10.3390/ijms222111595 PMC8583966

[55] A. Ciechanover, The ubiquitin-proteasome proteolytic pathway. Cell **79**, 13–21 (1994). 7923371 10.1016/0092-8674(94)90396-4

[56] X. Li , Ubiquitin- and ATP-independent proteolytic turnover of p21 by the REGgamma-proteasome pathway. Mol. Cell **26**, 831–842 (2007). 17588518 10.1016/j.molcel.2007.05.028

[57] X. Li , The SRC-3/AIB1 coactivator is degraded in a ubiquitin- and ATP-independent manner by the REGgamma proteasome. Cell **124**, 381–392 (2006). 16439211 10.1016/j.cell.2005.11.037

[58] X. Chen, L. Barton, Y. Chi, B. Clurman, J. Roberts, Ubiquitin-independent degradation of cell-cycle inhibitors by the REGgamma proteasome. Mol. Cell Proteomics **26**, 843–852 (2007). 10.1016/j.molcel.2007.05.022 PMC2031223 17588519

[59] H. Wu, J. Arron, TRAF6, a molecular bridge spanning adaptive immunity, innate immunity and osteoimmunology. Bioessays **25**, 1096–1105 (2003). 14579250 10.1002/bies.10352

[60] M. Chellaiah, L-Plastin phosphorylation: Possible regulation by a TNFR1 signaling cascade in osteoclasts. Cells **10**, 2432 (2021). 34572081 10.3390/cells10092432 PMC8464874

[61] B. Wong , TRANCE, a TNF family member, activates Akt/PKB through a signaling complex involving TRAF6 and c-Src. Mol. Cell **4**, 1041–1049 (1999). 10635328 10.1016/s1097-2765(00)80232-4

[62] J. Luo , LGR4 is a receptor for RANKL and negatively regulates osteoclast differentiation and bone resorption. Nat. Med. **22**, 539–546 (2016). 27064449 10.1038/nm.4076

[63] C. Wu , NLRP11 attenuates Toll-like receptor signalling by targeting TRAF6 for degradation via the ubiquitin ligase RNF19A. Nat. Commun. **8**, 1977 (2017). 29215004 10.1038/s41467-017-02073-3 PMC5719394

[64] M. Inomata, S. Niida, K. Shibata, T. Into, Regulation of Toll-like receptor signaling by NDP52-mediated selective autophagy is normally inactivated by A20. Cell Mol. Life Sci. **69**, 963–979 (2012). 21964925 10.1007/s00018-011-0819-y PMC3285758

[65] L. Li, REGγ is essential to maintain bone homeostasis by degrading TRAF6, preventing osteoporosis. ProteomeXchange. https://www.iprox.cn/page/project.html?id=IPX0010088000. Deposited 27 October 2024. 10.1073/pnas.2405265121 PMC11588133 39536082

