# Supplementary material for: REGγ is essential to maintain bone homeostasis by degrading TRAF6, preventing osteoporosis
Source: Proc Natl Acad Sci U S A. 2024 Nov 13;121(47):e2405265121. doi: 10.1073/pnas.2405265121 (PMC11588133; doi:10.1073/pnas.2405265121)
Supplement: Supplementary file 1 — Appendix 01 (PDF) [file pnas.2405265121.sapp.pdf]

PNAS

Supporting Information for

REGγ is essential to maintain bone homeostasis by degrading TRAF6, preventing osteoporosis.

Yingying Du[a,1], Hui Chen[b,c,d,1] Lei Zhou[e,1], Qunfeng Guo[f,1], Shuangming Gong[a], Siyuan Feng[a], Qiujing Guan[a], Peilin Shi[a], Tongxin Lv[a], Yilan Guo[g], Cheng Yang[f], Peng Sun[g], Kun Li[h,2], Shuogui Xu[i,2], and Lei Li [c,d, j,k,2]

[1]Y.D., H.C., L.Z. and Q. G. contributed equally to this work.

[2]To whom correspondence may be addressed.

Lei Li lli@bio.ecnu.edu.cn

Shuogui Xu shuogui_xu@smmu.edu.cn

Kun Li kunli12345@163.com

[a] Shanghai Key Laboratory of Regulatory Biology, Institute of Biomedical Sciences, School of Life Sciences, East China Normal University, Shanghai 200241, China.

[b] Department of Trauma-Emergency & Critical Care Medicine, Shanghai Fifth People's Hospital, Fudan University, Shanghai 200240, China.

[c] Joint Center for Translational Medicine, Shanghai Fifth People's Hospital, Fudan University and School of Life Science, East China Normal University, Shanghai 200241, China.

[d] School of Life Sciences, East China Normal University, Shanghai 200241, China.

[e] Department of Orthopedics, Shanghai General Hospital, Shanghai Jiao Tong University School of Medicine, Shanghai 200080, China.

[f] Department of Orthopedics, Changzheng Hospital, Naval Medical University, Shanghai 200003, China.

[g] The Key Laboratory of Adolescent Health Assessment and Exercise Intervention of the Ministry of Education, East China Normal University, Shanghai 200241, China.

[h] Health Science Center, East China Normal University, Shanghai 200241, China.

[i] Department of Emergency and Trauma, the First Affiliated Hospital of Naval Medical University, Shanghai 200433, China.

[j] Chongqing Key Laboratory of Precision Optics, Chongqing Institute of East China Normal University, Chongqing 401120, China.

[k] Shanghai frontiers science center of genome editing and cell therapy, East China Normal University, Shanghai 200241, China.

This PDF file includes:

Supporting text
Figures S1 to S8
Tables S1 to S4

Supporting Information Text

Materials and Methods

Human Samples. The human samples used in this study were provided by Shanghai General Hospital, including clinical data from both control and osteoporosis patients. The patients whose samples we collected have been excluded from those who have had fragile fractures within the past 6 months, those with bone tumors, those with secondary osteoporosis, those who have taken medication for osteoporosis, and those who have been bedridden for more than 3 months recently.

Plasmids. The pSG5-HA-REGγ constructs previously generated were used in this study. Based on the Homo sapiens *TRAF6* sequence, PCDH-SFB-*TRAF6*-FL, PCDH-SFB-*TRAF6*-N, PCDH-SFB-*TRAF6*-RZ$_{1}$, PCDH-SFB-*TRAF6*-RZ$_{1-2}$, PCDH-SFB-*TRAF6*-RZ$_{1-3}$, PCDH-SFB-*TRAF6*-C constructs were generated. Primers were listed in *SI Appendix*, Table S3.

Micro-CT Assay. 3D micro-CT analysis was performed according to the manufacturer's instructions. The femurs of mice were selected for X-ray microtomography (Skyscan 1076, Bruker) with a pixel size of 9 μm, and the region of interest of the distal femur below the growth plate was uniformly selected for reconstruction. 3D analysis and BMD analysis were performed using CTAnalyser software (Bruker). 3D reconstruction mapping was adjusted using CTVox software (Bruker).

Immunohistochemistry and Immunofluorescence Assays. For Immunohistochemistry assay, Mouse femurs were fixed with 4% paraformaldehyde for 2 days and were then decalcified in 0.5 M EDTA solution for 14 days, and the solution was changed every day. Gradient dehydration and paraffin embedding were performed after the femur tissue was completely softened. Six-μm sections were cut in a microtome (Leica, Germany) and then stained with hematoxylin-eosin (H&E) and TRAP staining.

For Immunofluorescence assay, BMMs and osteoclasts were fixed with 4% paraformaldehyde for 20 minutes, permeabilized with 0.1% Triton X-100 for 20 minutes, repaired with PBST for 30 minutes, and then blocked with 5% BSA for 1 hour. Cells were then incubated overnight at 4°C with 1:250 anti-TRAF6 and anti-REGγ. Subsequently, they were incubated with goat anti-mouse Alexa Fluor 488 secondary antibody and anti-rabbit Alexa Fluor 549 secondary antibody for 1 hour, followed by 5 minutes of incubation with DAPI. Observation and photography were performed under the two-photon laser confocal microscope.

In Vitro Osteoclast Differentiation Assay. Bone marrow cells isolated from femurs of 6-8 weeks mice were cultured overnight in α-MEM medium with 10% fetal bovine serum (FBS). The non-adherent cells were transferred to another plate with 10ng/mL M-CSF for 24 hours.

Subsequently, BMMs were digested and seeded at a density of 1 × 10^5/mL in the same medium for further culture, and the next day was changed to a differentiating medium (α-MEM + 10% FBS + 10 ng/mL M-CSF + 50 ng/mL RANKL). The differentiation medium was changed every two days, for a total of 6 days.

In Vitro Osteoblast Differentiation Assay. As previously described, bone marrow cells isolated from femurs of 6-8 weeks mice were cultured overnight in α-MEM medium with 10% fetal bovine serum (FBS). Following this, the medium was replaced, and the adherent cells were mesenchymal stem/progenitor cells (MSCs). For osteoblast differentiation, MSCs from the second or third passage were typically utilized. 5 × 10^4 MSCs were plated per well in 12-well plates and cultured in osteoblast differentiating medium (10 nM dexamethasone, 10 mM β-glycerophosphate, and 50 μM ascorbic acid in α-MEM containing 10% FBS) for 7/14/21 days with medium changes every three days.

TRAP Staining Assay. After fixation, permeabilization, and repair of cells and deparaffinized tissue slices, TRAP staining was performed using a TRAP staining kit according to the manufacturer's instructions. Briefly, cells were stained with the solution at 37°C for 60 minutes, followed by washing with PBS to remove excess staining solution. TRAP-positive osteoclasts with five or more nuclei were counted during imaging. Tissue slices were then counterstained with 2% methyl green staining solution for 2 minutes after TRAP staining, rinsed with PBS, and mounted with neutral resin.

Pit Formation Assay. Mature osteoclasts were digested with Versene and then inoculated onto bone slices and cultured for 24 hours, followed by dehydration in graded alcohol. Finally, the bone slices were placed under the two-photon laser confocal microscope (Leica TCS SP8) to observe and record the morphology of the phagocytic pits.

ALP Staining，Alizarin Red S Staining and Von Kossa Staining. ALP staining and Alizarin Red S staining were performed using the BCIP/NBT Alkaline Phosphatase Color Development Kit and Alizarin Red S Kit following the manufacturers’ protocols. For Von Kossa staining, cells were fixed with 4% paraformaldehyde solution, rinsed with deionized water, and then stained with 1% silver nitrate solution for 20 minutes under UV light. Subsequently, cells were incubated with 5% sodium thiosulfate for 5 minutes.

RNA Extraction and Real-Time Quantitative PCR Assays. Total RNA was extracted using TRIzol reagent-phenol chloroform. RNA was transcribed to cDNA using 5 × HiScript II qRT SuperMix. qRT-PCR was performed using 2 × ChamQ Universal SYBR qPCR Master Mix. 18S was used for relative expression level quantification. Fold changes were calculated by the $2^{-\Delta\Delta Ct}$ method. The primers used in this study showed in *SI Appendix*, Table S3.

Western Blotting Assay. The cells were resuspended in lysis buffer and protein separated by 8%-12% SDS-gel. The separated proteins were then transferred to nitrocellulose membranes and incubated with primary antibodies overnight at 4 °C. The fluorescent-labeled secondary antibody (1:5000 dilutions) is then combined with the primary antibody and analyzed in the LI-COR Odyssey Infrared Imaging System. The primary antibodies used in this study showed in the Cell regent or Resource.

Co-immunoprecipitation Assay. The cells were harvested and lysed with 1% NP-40 buffer (25 mM Tris-HCl, pH 7.4, 150 mM NaCl, 1% protease inhibitors, 1% NP-40) at 4 °C after plasmids transfection for 36 hours. After the lysis is completed, centrifuge at high speed at 4 °C for 20 minutes, and incubate the collected supernatant with Flag beads for 4 hours. Flag beads bind to Flag-tagged proteins, respectively. The pellet was washed 3 times with washing buffer and subjected to immunoblot analysis.

In Vitro Proteolytic Analysis Assay. The vitro proteolytic system includes 5 μL TRAF6 protein, 0.25 μg 20S proteasome, and 2 μg REGγ heptameric, and the total reaction system is 50 μL. TRAF6 protein was translated by TNT® T7 Quick Coupled Transcription /Translation System (Promega). The above reaction system was incubated at 30 °C for 3 hours and results were analyzed by Western blotting.

Enzyme-Linked Immunosorbent Assay (ELISA). The levels of REGγ in mouse serum, as well as the levels of REGγ, C-terminal telopeptide of type I collagen (CTX-1), and N-terminal propeptide of type 1precollagen (P1NP) in human serum, were measured using corresponding ELISA kits. The concentrations of CTX-1, P1NP, and REGγ were determined using standard curves.

Statistics. All results are presented as the mean with SD unless notified specifically. Statistics were assessed using a two tailed student t-test or one-way ANOVA analysis. Markers of significance are as follows: N.S., $p > 0.05$; *, $P < 0.05$; **, $P < 0.01$; ***, $P < 0.001$.

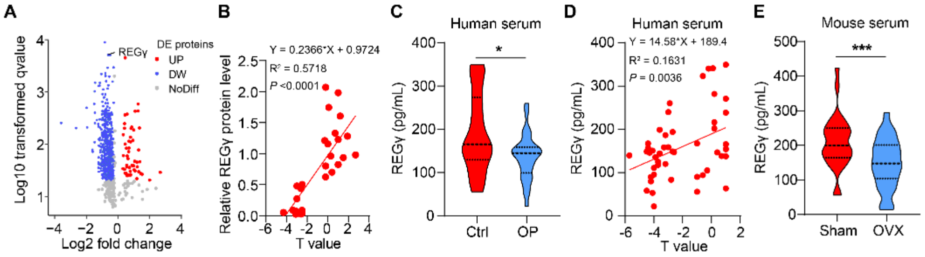

Fig. S1. REGγ is identified as a potential biomarker in osteoporosis.

(A) Volcano plots of proteins in bone specimens of Ctrl and OP human samples. The red dots represent proteins that were upregulated at least 1.2 times in bone specimens, and the green dots represent proteins that were downregulated at least 0.83 times in bone specimens (n = 4). Control: Ctrl; osteoporosis: OP.

(B) The correlation between REGγ protein expression level in Ctrl and OP samples and BMD.

(C) The REGγ concentration in Ctrl (n = 22) and OP (n = 28) populations.

(D) The correlation between REGγ levels of the serum from Ctrl (n = 22) and OP (n = 28) populations and BMD.

(E) The REGγ concentration in sham (n = 27) and OVX (n = 55) mouse serum detected by Elisa assay.

Markers of significance are as follows: P < 0.05; ***, P < 0.001.

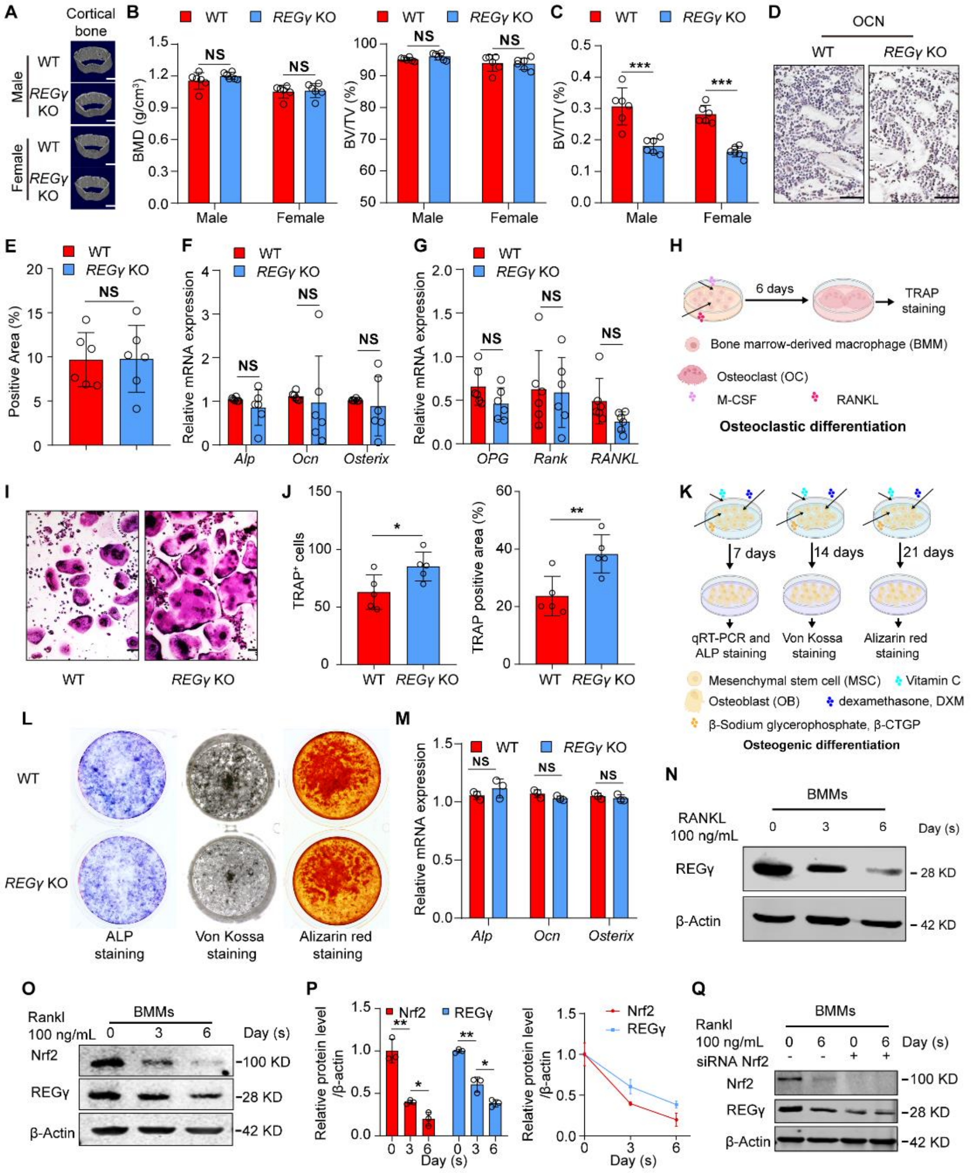

Fig. S2. REGγ regulates bone mass by modulating osteoclasts.

(A) Representative micro-CT images showing the cortical bone of femurs from 6-month-old WT littermates and *REGγ* KO mice (similar results were obtained in all mice, n = 6). Scale bar: 1 mm.

(B) Micro-CT measurements of BMD and BV/TV in femurs from 6-month-old WT littermates and *REGγ* KO mice. BMD, bone mineral density; BV/TV, bone volume as a fraction of total bone volume (n = 6).

(C) The BV/TV quantification in the femurs of 6-month-old WT littermates and *REGγ* KO mice was conducted using H&E staining.

(D) Representative osteocalcin (OCN) IHC staining in femurs of 6-month-old REGγ KO female mice and WT littermates. Scale bar: 50 μm.

(E) Quantification of OCN positive area in (D) (n = 6).

(F) Quantification of *Alp, Ocn* and *Osterix* expression in femurs of 6-month-old *REGγ* KO mice and WT littermates (n = 6)

(G) Quantification of *OPG*, *Rank*, and *RANKL* levels in femurs of 6-month-old REGγ KO mice and WT littermates (n = 6)

(H) Schematic illustration depicting the differentiation process of bone marrow-derived macrophages (BMMs) into osteoclasts: BMMs were treated with 10ng/mL M-CSF and 50 ng/mL RANKL, and induced for differentiation over a period of 6 days.

(I) Representative TRAP staining of osteoclasts from WT and *REGγ* KO BMMs. Scale bar: 100 μm.

(J) Quantification of osteoclast number and TRAP-positive area in (H) (n = 5).

(K) Schematic diagram illustrating the process of differentiation of bone marrow mesenchymal stem cells (MSCs) into osteoclasts: MSCs were exposed to 10 nM dexamethasone, 100 mM β-glycerophosphate, and 50 μM ascorbic acid, and induced for differentiation over a period of 7/14/21 days.

(L) Representative ALP staining, Alizarin Red S staining and Von Kossa staining of osteoblasts from WT littermates and *REGγ* KO mice (n = 3).

(M) Quantification of *Alp*, *Ocn*, and *Osterix* expression in WT and *REGγ* KO osteoblasts (n = 3).

(N) Western blot analysis of REGγ protein levels in BMMs treated with RANKL at different time points (0, 3, 6 days). three times independently repeated experiments.

(O) Western blot analysis of Nrf2 and REGγ protein levels in BMMs treated with RANKL at different time points (0, 3, 6 days). three times independently repeated experiments.

(P) Quantification of Nrf2 and REGγ protein levels as shown in (O).

(Q) Western blot analysis of Nrf2 and REGγ protein levels in BMMs treated with siRNA and RANKL. Line 1: BMMs. Line 2: BMMs treated with RANKL for 6 days to induce differentiation. Line 3: BMMs transfected with siRNA Nrf2 for 2days to silence Nrf2 expression. Line 4: BMMs transfected with siRNA Nrf2 for 2days, followed by treatment with RANKL for 6 days to induce differentiation. siRNA Nrf2 target sequence: 5′- CCCGAATTACAGTGTCTTAAT -3′. Three times independently repeated experiments.

Markers of significance are as follows: N.S, $p > 0.05$; *, $P < 0.05$; **, $P < 0.01$; ***, $P < 0.001$.

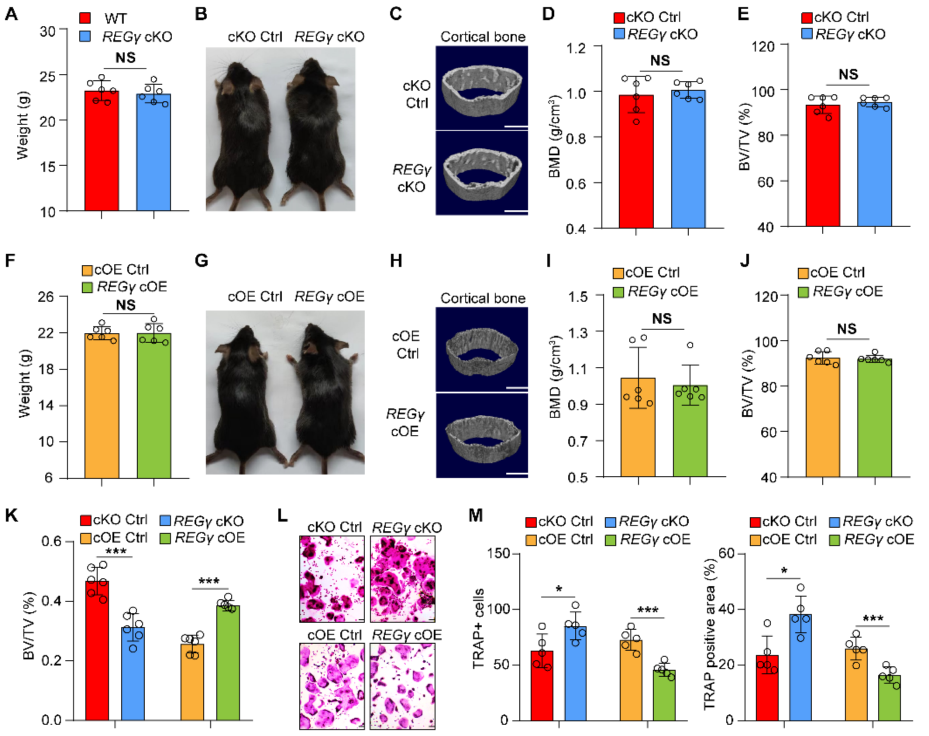

Fig. S3 REGγ suppresses RANKL-induced osteoclastogenesis.

(A) The body weights of 2-month-old cKO Ctrl and *REGγ* cKO mice.

(B) Representative images of cKO Ctrl and *REGγ* cKO mice at 2 months.

(C) Representative micro-CT images showing the cortical bone of femurs from 2-month-old Ctrl and *REGγ* cOE mice (similar results were obtained in all mice, n = 6). Scale bar: 1 mm (all panels).

(D-E) Micro-CT measurements of BMD and BV/TV in femurs from 2-month-old Ctrl and *REGγ* cOE mice. (n = 6).

(F) The body weights of 2-month-old cOE Ctrl and *REGγ* cOE mice.

(G) Representative images of cOE Ctrl and *REGγ* cOE mice at 2 months.

(H) Representative micro-CT images showing the cortical bone of femurs from 2-month-old Ctrl and *REGγ* cOE mice (similar results were obtained in all mice, n = 6). Scale bar: 1 mm (all panels).

(I-J) Micro-CT measurements of BMD and BV/TV in femurs from 2-month-old Ctrl and *REGγ* cOE mice (n = 6).

(K) The BV/TV quantification in the femurs of 2-month-old cKO Ctrl, *REGγ* cKO, cOE Ctrl and *REGγ* cOE mice was conducted using H&E staining (n = 6).

(L) Representative TRAP staining of osteoclasts from cKO Ctrl, *REGγ* cKO, cOE Ctrl and *REGγ* cOE BMMs. Scale bar: 100 μm.

(M) Quantification of osteoclast number and TRAP-positive area in (L) (n = 5).

Markers of significance are as follows: N.S, $p > 0.05$; *, $P < 0.05$; **, $P < 0.01$; ***, $P < 0.001$. Control: Ctrl; *REGγ* cKO: bone marrow-derive macrophage (BMM)-specific *REGγ* KO; *REGγ* cOE: bone marrow-derive macrophage (BMM)-specific *REGγ* OE.

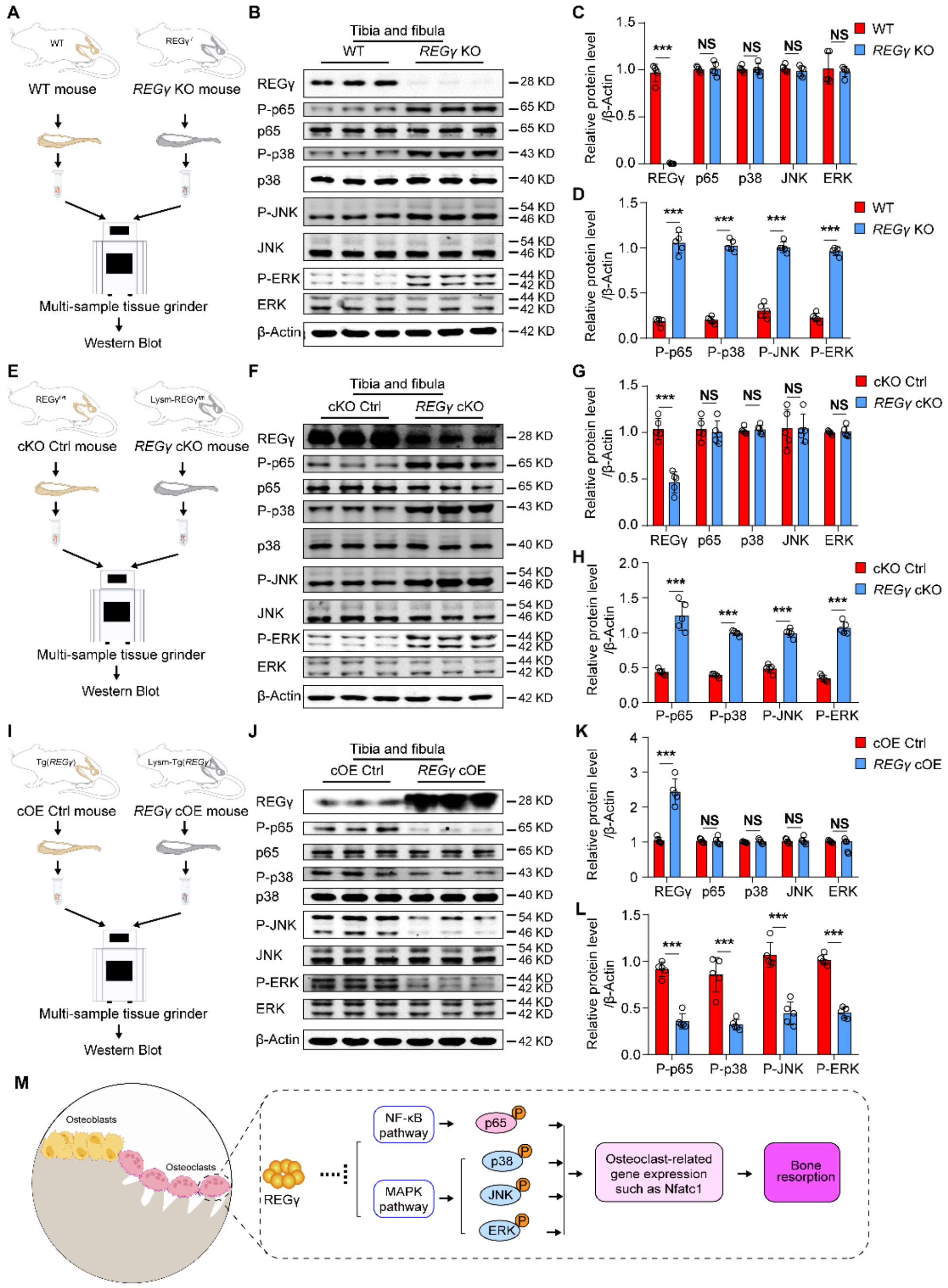

Fig. S4. REGγ specifically inhibits osteoclast activity by suppressing the NF-κB and MAPK signaling pathways.

(A) Schematic diagram of bone tissue protein extraction process: Tibia and fibula from both 6-month-old WT and *REGγ* KO mice are collected and homogenized using a tissue homogenizer. After thorough lysis, proteins are extracted for Western blotting detection.

(B-D) Western blotting images illustrating alterations in the MAPK and NF-κB signaling pathways in bone tissues of WT and *REGγ* KO mice. The above results were analyzed using ImageJ (C, D) (n = 5).

(E) Schematic diagram of bone tissue protein extraction process: Tibia and fibula from both 2-month-old cKO Ctrl and *REGγ* cKO mice are collected and homogenized using a tissue

homogenizer. After thorough lysis, proteins are extracted for Western blotting detection.

(F-H) Western blotting images illustrating alterations in the MAPK and NF-κB signaling pathways in bone tissues of cKO Ctrl and *REGγ* cKO mice. The above results were analyzed using ImageJ (G, H) (n = 5).

(I) Schematic diagram of bone tissue protein extraction process: Tibia and fibula from both 2-month-old cOE Ctrl and *REGγ* cOE mice are collected and homogenized using a tissue homogenizer. After thorough lysis, proteins are extracted for Western blotting detection.

(J-L) Western blotting images illustrating alterations in the MAPK and NF-κB signaling pathways in bone tissues of cKO Ctrl and *REGγ* cKO mice. The above results were analyzed using ImageJ (K, L) (n = 5).

(M) Schematic diagram of bone resorption mechanism in WT mice: REGγ inhibits the activation of MAPK and NF-κB signaling pathways, thereby inhibiting the occurrence of osteoporosis.

Markers of significance are as follows: N.S, $p > 0.05$; *, $P < 0.05$; **, $P < 0.01$; ***, $P < 0.001$.

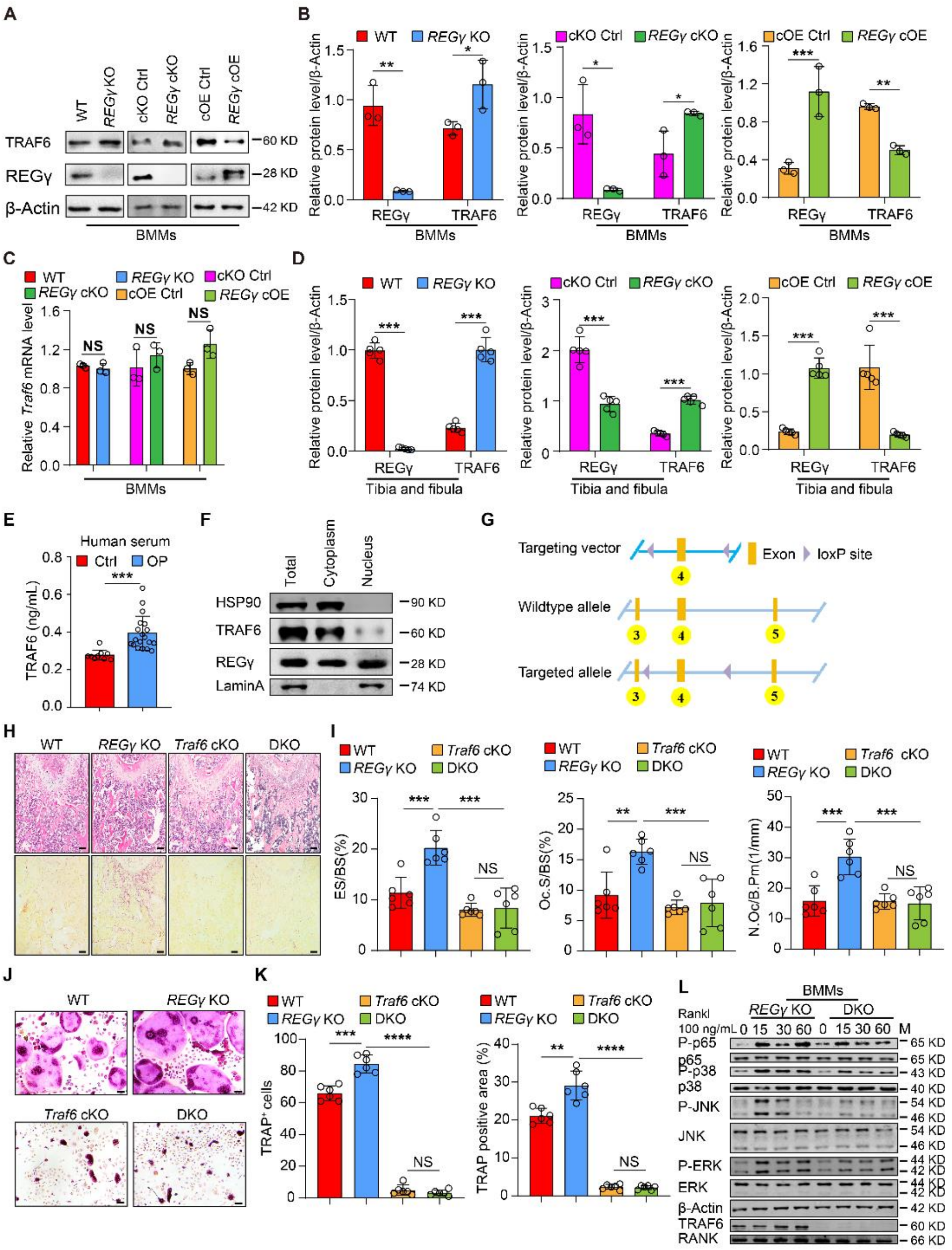

Fig. S5. REGγ increased bone mass through ubiquitin-independent degradation of TRAF6.

(A-B) Western blotting analysis of TRAF6 protein levels in WT, *REGγ* KO, cKO Ctrl, *REGγ* cKO, cOE Ctrl and *REGγ* cOE hindlimb bones (A), with quantification using ImageJ (B) (n =3).

(C) Quantification of Traf6 mRNA level in BMMs of 2-month-old *REGγ* KO, *REGγ* cKO, *REGγ* cOE mice and their WT littermates (n =3).

(D) Quantification of REGγ and TRAF6 protein levels in hindlimb bones of WT, *REGγ* KO, cKO Ctrl, *REGγ* cKO, cOE Ctrl, and *REGγ* cOE mice (n =5).

(E) The plasma TRAF6 concentration in Ctrl (n = 9) and OP (n = 19) human serum.

(F) Western blot analysis of REGγ and TRAF6 protein levels in the nuclear and cytoplasmic fractions of WT BMMs, three times independently repeated experiments.

(G) Schematic diagram of *Traf6*$^{fl/fl}$ mouse construction strategy.

(H) Representative images of H&E staining and TRAP staining of femurs from WT, *REGγ* KO, *Traf6* cKO and DKO mice. Scale bar: 100 μm.

(I) Histomorphometrical analysis of TRAP staining in (H) (n = 6). Scale bar: 100 μm.

(J) Representative TRAP staining of osteoclasts from WT, *REGγ* cKO, *Traf6* cKO and DKO BMMs. Scale bar: 100 μm.

(K) Quantification of osteoclast number and TRAP-positive area in (J) (n = 6).

(L) Western blotting images depict alterations in TRAF6, RANK, as well as the MAPK and NF-κB signaling pathways in *REGγ* KO and DKO BMMs during RANKL stimulation. three times independently repeated experiments.

Markers of significance are as follows: N.S, $p > 0.05$; *, $P < 0.05$; **, $P < 0.01$; ***, $P < 0.001$.

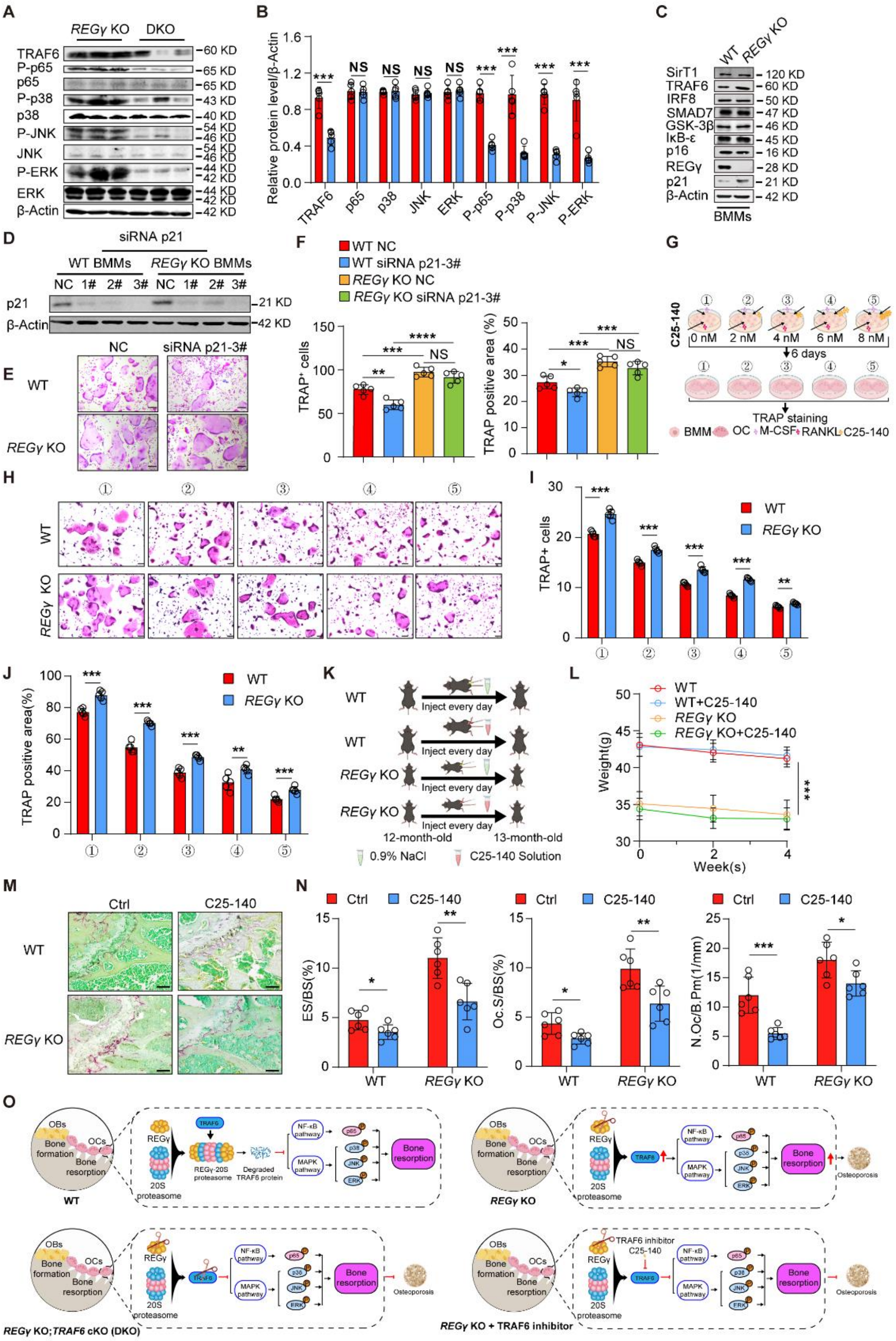

Fig. S6. REGγ increased bone mass through ubiquitin-independent degradation of TRAF6.

(A-B) Western blotting images depict alterations in TRAF6, MAPK and NF-κB signaling pathways in the hindlimb bones of *REGγ* KO and DKO mice (A), with quantification using ImageJ (B) (n = 5).

(C) Western blotting images showing the protein levels of SirT1, TRAF6, IRF8, SMAD7, GSK-3β, IκBε, p16, p21, REGγ in WT and *REGγ* KO BMMs, three times independently repeated experiments.

(D) Western blot analysis of p21 protein levels in WT and *REGγ* KO BMMs treated with siRNA p21. BMMs transfected with siRNA p21 for 2days to silence p21 expression. siRNA p21-1# target sequence: 5′- CCCGAGAACGGTGGAACTT -3′. siRNA p21-2# target sequence: 5′-GGAGCAAAGTGTGCCGTTG-3′. siRNA p21-3# target sequence: 5′-CAATCCTGGTGATGTCCGA-3′. Three times independently repeated experiments.

(E) Representative TRAP staining of BMMs from WT and REGγ KO mice treated with or without siRNA p21-3#. WT and *REGγ* KO BMMs transfected with siRNA p21 for 2days, followed by treatment with RANKL for 6 days to induce differentiation. Scale bar: 250 μm.

(F) Quantification of osteoclast number and TRAP-positive area in (E), n=5.

(G) Schematic illustration depicting osteoclast differentiation of BMMs under varying concentrations of C25-140: BMMs were treated with 10 ng/mL M-CSF and 50 ng/mL RANKL to induce osteoclast differentiation over a period of 6 days. During induction differentiation, the concentrations of C25-140 used were 0,2 nM,4 nM,6nM and 8nM.

(H) Representative TRAP staining of osteoclasts from WT and *REGγ* KO BMMs treated with various concentrations of C25-140. Scale bar: 100 μm.

(I) Quantification of osteoclast number in (H) (n=5).

(J) Quantification of TRAP-positive area in (H) (n=5).

(K) Schematic diagram illustrating the experimental design of C25-140 treatment in WT and *REGγ* KO mice: WT and REGγ KO mice were injected with saline as the control group, and injected with C25-140 as the experimental group. Injections were administered intraperitoneally daily for one month.

(L) Weight changes in WT and *REGγ* KO mice during C25-140 treatment.

(M) Representative immunofluorescence images of TRAP expression in 12-month-old WT, *REGγ* KO, C25-140-treated WT and C25-140-treated *REGγ* KO mice (similar results were obtained in all mice, n = 6). Scale bar: 50 μm.

(N) Histomorphometrical analysis of TRAP staining in (M) (n = 6).

(O) Schematic diagram illustrating the bone resorption mechanisms in WT, *REGγ* KO, DKO and C25-140-treated *REGγ* KO mice.

Markers of significance are as follows: N.S, $p > 0.05$; *, $P < 0.05$; **, $P < 0.01$; ***, $P < 0.001$; ****, $P < 0.0001$.

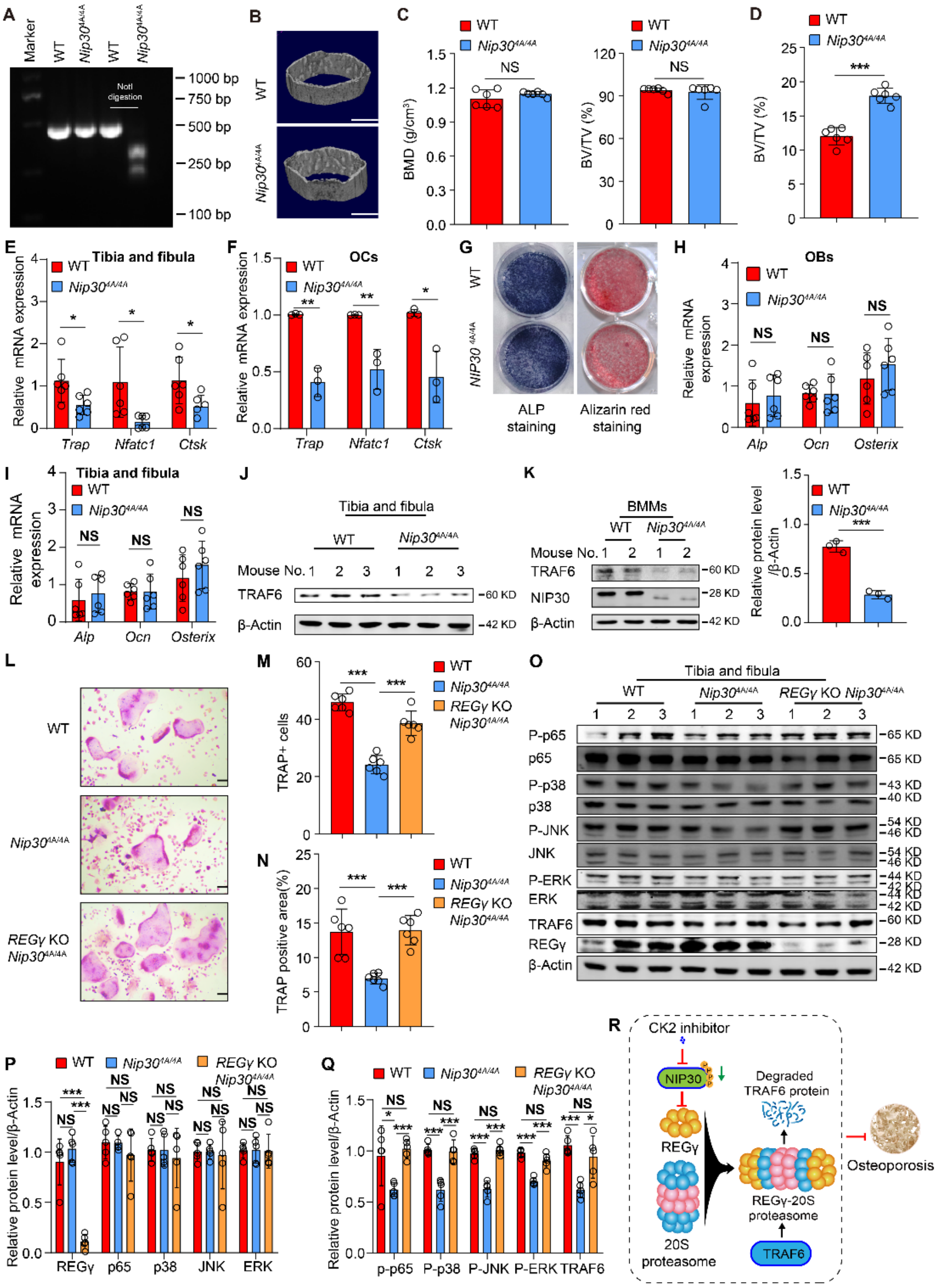

Fig. S7. NIP30 dephosphorylation activates the ubiquitin-independent degradation of TRAF6 to alleviate osteoporosis.

(A) Genotype identification results of WT and *Nip30*$^{4A/4A}$ mice.

(B) Representative micro-CT images showing the cortical bone of the femurs from 2-month-old WT and *Nip30*$^{4A/4A}$ mice (similar results were obtained in all mice). Scale bar: 1 mm.

(C) Micro-CT measurements of BMD and BV/TV in femurs from 2-month-old WT and *Nip30*$^{4A/4A}$ mice (n = 6).

(D) The BV/TV quantification in the femurs of 2-month-old WT and *Nip30*$^{4A/4A}$ mice was conducted using H&E staining (n = 6).

(E) Quantification of *Trap*, *Nfatc1*, and *Ctsk* expression in the tibia and fibula of *Nip30*$^{4A/4A}$ mice and WT littermates (n = 6).

(F) Quantification of *Trap*, *Nfatc1*, and *Ctsk* expression in WT and *Nip30*$^{4A/4A}$ osteoclasts, three times independently repeated experiments.

(G) Representative ALP staining and Alizarin Red S staining of osteoblasts from WT and *Nip30*$^{4A/4A}$ mice.

(H) Quantification of *Alp*, *Ocn*, and *Osterix* expression in WT and *Nip30*$^{4A/4A}$ osteoblasts , three times independently repeated experiments.

(I) Quantification of *Alp*, *Ocn*, and *Osterix* expression in the tibia and fibula of WT and *Nip30*$^{4A/4A}$ mice (n = 6).

(J) Western blotting analysis of TRAF6 protein levels in WT and *Nip30*$^{4A/4A}$ hindlimb bones.

(K) Western blotting analysis of TRAF6 protein levels in WT and *Nip30*$^{4A/4A}$ BMMs, with quantification using ImageJ, three times independently repeated experiments.

(L) Representative TRAP staining of osteoclasts from WT, *Nip30*$^{4A/4A}$ and *REGγ* KO *Nip30*$^{4A/4A}$ BMMs treated with RANKL stimulation. Scale bar: 100 μm.

(M) Quantification of osteoclast number in (L) (n=6).

(N) Quantification of TRAP-positive area in (L) (n=6).

(O-Q) Western blotting images depict alterations in REGγ, TRAF6, as well as the MAPK and NF-κB signaling pathways in the hindlimb bones of WT, *Nip30*$^{4A/4A}$ and *REGγ* KO *Nip30*$^{4A/4A}$ mice (O). The above results were analyzed using ImageJ (P, Q) (n = 5).

(R) Hypothesized Schematic Diagram of CK2 Inhibitor-Mediated Suppression of NIP30 in Osteoporosis Regulation.

Markers of significance are as follows: N.S, $p > 0.05$; *, $P < 0.05$; **, $P < 0.01$; ***, $P < 0.001$.

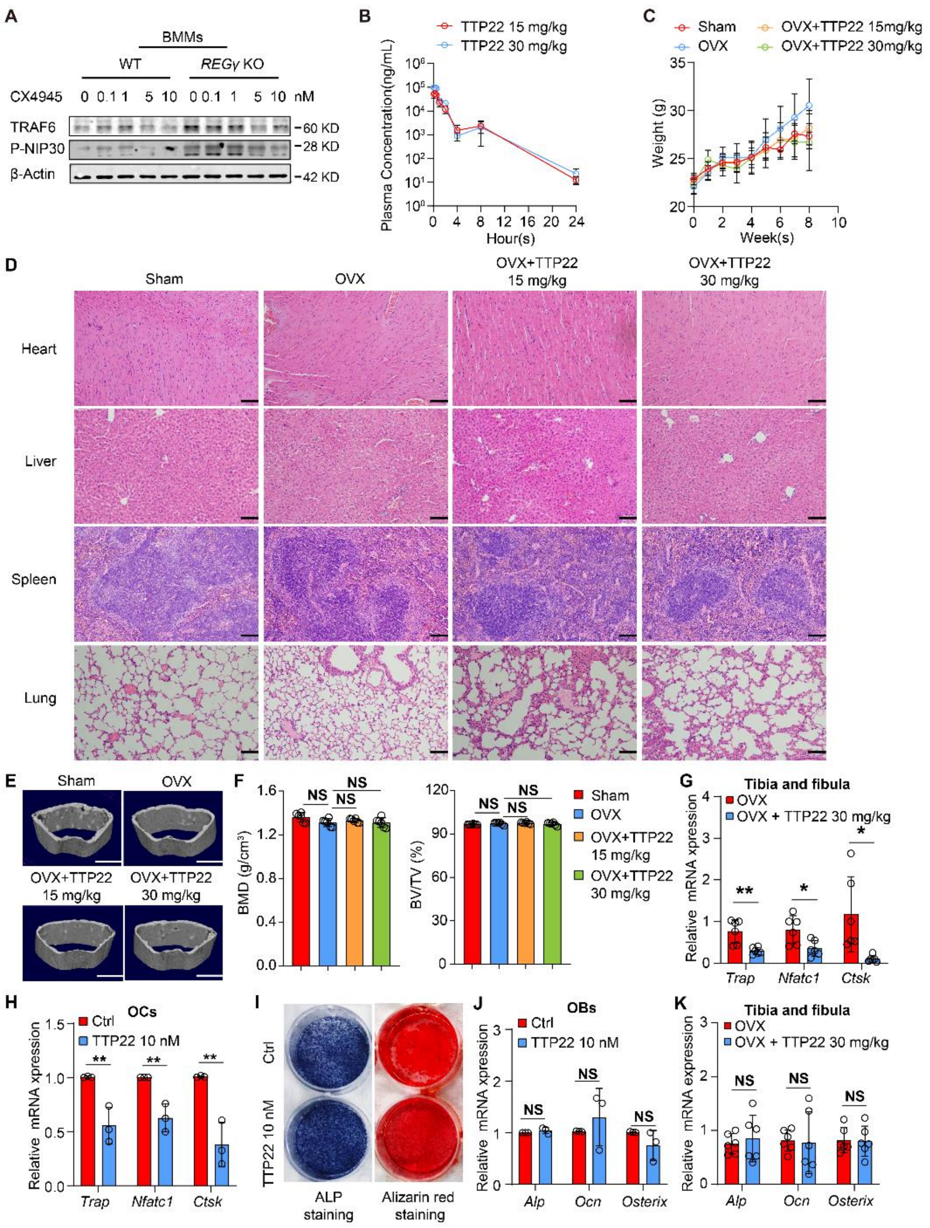

Fig.S8. TTP22 alleviates osteoporosis by regulating the CKII/NIP30/REGγ/TRAF6 axis.

(A) Western blotting analysis of the protein levels of P-NIP30 and TRAF6 in BMMs with or without CX4945 treatment for 24 hours.

(B) Pharmacokinetic analysis of TTP22 in mice. Dosing: 15 mg/kg and 30 mg/kg.

(C) The weight changes in sham, OVX, OVX+15 mg/kg and OVX+30 mg/kg mice during TTP22 treatment.

(D) Representative images of H&E staining of purtenance of 5-month-old sham, OVX, OVX+15 mg/kg and OVX+30 mg/kg mice (similar results were obtained in all mice, n = 6). Scale bar: 100 μm.

(E) Representative micro-CT images showing the cortical bone of femurs from 5-month-old sham, OVX, OVX+15 mg/kg and OVX+30 mg/kg mice (similar results were obtained in all mice).

Scale bar: 1 mm.

(F) Micro-CT measurements of BMD and BV/TV in femurs from 5-month-old sham, OVX, OVX+15 mg/kg and OVX+30 mg/kg mice (n = 6).

(G) Quantification of *Trap*, *Nfatc1*, and *Ctsk* expression in the tibia and fibula of WT and TTP22-treated WT mice (n = 6).

(H) Quantification of *Trap*, *Nfatc1*, and *Ctsk* expression in osteoclasts of WT and TTP22-treated WT mice, three times independently repeated experiments.

(I) Representative ALP staining and Alizarin Red S staining of osteoblasts from WT mice treated with or without TTP22, three times independently repeated experiments.

(J) Quantification of *Alp*, *Ocn*, and *Osterix* expression in osteoblasts from WT mice treated with or without TTP22, three times independently repeated experiments.

(K) Quantification of *Alp*, *Ocn*, and *Osterix* expression in the tibia and fibula of WT mice treated with or without TTP22 (n = 6).

Markers of significance are as follows: N.S, $p > 0.05$; *, $P < 0.05$; **, $P < 0.01$.

Table S1 The Information of control and osteoporosis patients

Group	Sex	Age （yr）	height （cm）	weight （Kg）	T-score	β-CTX	P1NP
OP-1	Female	71	168	85.4	-2.6	395	61.85
OP-2	Female	69	157	64.8	-2.8	805	91.66
OP-3	Female	69	150	62.0	-3.3	180	37.92
OP-4	Female	71	157	51.0	-3.8	370	51.33
Ctrl-1	Female	59	164	66.0	-1.7	158	24.80
Ctrl-2	Female	72	165	71.0	-1.1	173	33.01
Ctrl-3	Female	46	150	50.3	-1.1	498	52.48
Ctrl-4	Female	49	147	56.0	-0.7	805	53.15

Table S1. The information of control and osteoporosis patients.

Table S2 The Information of control and osteoporosis patients for Elisa Assay

Patient	Sex	Age (yr)	height (cm)	weight (Kg)	T-score	β-CTX	P1NP
1	male	33	176	108.3	1	338	18.39
2	Female	60	168	71.6	1	360	30.72
3	male	65	164	77.4	1	312	21.57
4	male	50	163	62.5	1	266	15.53
5	male	68	172	72.8	1	432	11.27
6	male	51	180	84.5	1	173	10.86
7	Female	20	155	53.2	0.5	325	3.40
8	male	64	172	80.0	-0.4	389	5.09
9	male	21	193	114.4	1	461	8.24
10	male	41	174	84.7	0.2	518	9.22
11	male	57	167	58.1	-1	449	3.87
12	male	64	164	69.1	1	392	5.55
13	male	67	170	72.4	-1	300	4.53
14	male	57	179	71.5	1	455	2.74
15	male	39	173	62.7	1	873	14.35
16	male	34	174	82.0	0.1	1117	11.48
17	male	80	166	62.3	-0.6	1012	9.84
18	Female	37	157	61.1	-0.2	840	19.87
19	Female	42	167	67.6	0.7	575	7.36
20	male	28	181	95.0	-0.3	598	5.65
21	male	73	171	69.7	0.2	1058	7.46
22	Female	64	159	67.0	-0.2	796	37.91
23	Female	66	151	59.0	-0.8	629	43.07
24	Female	72	149	59.6	-0.9	222	20.91
25	Male	60	157	60.9	-1.1	409	39.19
26	Female	58	158	58.6	-1.2	381	66.70
27	Female	74	165	65.0	-1.4	563	33.97
28	Female	78	148	58.7	-1.5	101	26.26
29	Female	65	155	70.0	-1.6	753	57.70
30	Female	53	165	67.4	1.9	407	20.79
31	Female	70	151	51.5	-3.8	133	38.15
32	Female	84	141	51.6	-4.3	284	26.77
33	Female	59	160	55.0	-3.7	222	22.33
34	Female	64	147	56.6	-3.5	527	64.40
35	Female	68	151	48.1	-3.0	80	18.40
36	Female	54	158	66.5	-2.9	1351	53.40
37	Female	68	152	72.0	-2.8	458	54.46
38	Female	73	157	56.7	-2.7	602	47.20
39	Female	65	154	69.6	-2.6	153	23.58
40	Female	75	150	63.8	-2.5	471	36.70
41	Female	76	154	53.7	-3.4	248	43.97
42	Female	67	158	67.5	-2.4	765	65.31
43	Female	78	150	55.0	-3.5	989	76.96
44	Male	69	164	72.5	-3.2	626	30.10
45	Female	66	155	50.0	-3.7	696	68.30
46	Female	52	154	56.9	-2.9	1410	109.50
47	Female	55	163	50.0	-3.2	218	49.15
48	Female	68	142	52.5	-3.2	615	65.54
49	Female	62	165	50.0	-3.4	865	67.30

50	Female	59	160.5	50.4	-2.9	204	21.41
51	Female	66	154	56	-3	165	23.24
52	Female	69	160	50	-5.7	829	69.97
53	Female	88	160	53	-3.7	684	67.81
54	Female	74	155.5	48.9	-4.1	934	117.96
55	Female	67	165	55	-4.2	449	44.07
56	Female	68	151.5	54.9	-3	815	87.92
57	Female	80	154.5	60.1	-3.2	1178	101.48
58	male	75	166	72.8	-3.3	566	75.33
59	male	75	150.5	55.8	-4	230	16.81
60	Female	68	155.5	68.1	-3.6	417	36.03
61	male	53	171.5	67.3	-2.7	696	66.32
62	Female	65	152.5	44.4	-3.1	693	74.34
63	Female	53	145	51.1	-2.6	512	50.58
64	male	71	163.5	67.2	-2.8	498	58.72
65	Female	61	160.5	62	-3.6	616	61.70
66	Female	79	155.5	55	-4.5	801	89.36
67	Female	70	152	58.8	-3.9	601	85.04
68	Female	61	162	71.1	-4.4	724	66.12
69	male	64	155.5	54.7	-4.4	579	58.43
70	Female	88	160	53	-3.7	684	67.81
71	Female	69	152	70	-4.3	703	78.49
72	Female	70	152.5	59	-4	734	75.43
73	Female	75	150.5	55.8	-4	644	61.67
74	Female	79	152	63.6	-3.7	605	63.17
75	Female	74	153.5	55	-4.2	663	45.52
76	Female	66	162	56.6	-4.1	638	55.64
77	Female	80	154	69.2	-4.5	759	68.51
78	Female	67	146	59.3	-3.2	856	69.22

Table S2. The information of control and osteoporosis patients for Elisa Assay.

Table S3 | Primers

Primers for qPCR

Gene	Forward Primer sequence	Reverse Primer sequence
18s	GGACACGGACAGGATTGACA	GACATCTAAGGGCATCACAG
mREGγ	TCCTCACCAATAGCCACG	CTCGATCAGCAGCCGAAT
mTraf6	GAGTTTGACCCACCTCTGGA	TTTCATTGTCAACTGGGCACT
mTrap	ACACAGTGATGCTGTGTGGCAACTC	CCAGAGGCTTCCACATATATGATGG
mNfatc1	GGGTCAGTGTGACCGAAGAT	GGAAGTCAGAAGTGGGTGGA
mCtsk	GGCCAACTCAAGAAGAAAAC	GTGCTTGCTTCCCTTCTGG
mAlp	TGGTTACTGCTGATCATTCCCACG	AATGTAGTTCTGCTCATGGACGCC
mOsteocalcin	AGTCACCAACCACAGCATCC	TTTGTCCCTTCCCTTCTGCC
mOsteorix	ATGGCGTCCTCTCTGCTTG	TGAAAGGTCAGCGTATGGCTT

Primers for Genotype Identification

Genotype	Primer name	Primer sequence
REGγ WT/KO	Common	CACGATGGACTGGATGGT
	Wildtype	CTAACATAACTTACCTTGCC
	Knockout	TCGAGCGAGCACGTACT
REGγ $^{fl/fl}$	REGγ-loxPtF1	AATTTCAAGGTGAGGGCGAGACAG
	REGγ-loxPtR1	AATAAACGTGGGACAGTCCCTCACT
	REGγ-loxPtF2	ACCAGCTCAGGAGGTAAGGTCCAAT
	REGγ-loxPtR2	CACAATTCTAAGTGACTCCACCCCC
LSL-*REGγ*	REGγ-OE1	CAGACTTGTGGGATACAGAAGAC
	REGγ-OE2	AGTCCACCTCACTCCTCATAAC
	REGγ-OE3	GGTTGGCTATAAAGAGGTCATCAG
Lysm -cre	Lysm-cre Mutant	CCCAGAAATGCCAGATTACG
	Lysm-cre Common	CTTGGGCTGCCAGAATTTCTC
	Lysm-cre Wildtype	TTACAGTCGGCCAGGCTGAC
Traf6 $^{fl/fl}$	Traf6-loxPtF1	GAAATTAGATTCAGGGATCACCC
	Traf6-loxPtR1	GAGGTCAGAAGCATGGAAGT
	Traf6-loxPtF2	GAGTGACCTTTTCAGCACAGTTTA
	Traf6-loxPtR2	CGGTGACTGAAGTCCACGTT
Nip30 $^{4A/4A}$	NIP30-F	TGGGAATGGCCCTTCAGCATT
	NIP30-R	ATCAATGGGACAGGTTTGGTCATCT

Primers for Plasmid Construction

Plasmid	Forward Primer sequence	Reverse Primer sequence
PCDH-SFB-*TRAF6*-FL	CAGCGGGAGCCCGGATCCATGAGTCTGCTAAAC	CAGCGGGAGCCCGGATCCATGAGTCTGCTAAAC
PCDH-SFB-*TRAF6*-N	CAGCGGGAGCCCGGATCCATGAGTCTGCTAAAC	CAGATCCTTCGCGGCCGCCTATCGTTTGAGCTCACT
PCDH-SFB-TRAF6-RZ_1	CAGCGGGAGCCCGGATCCATGAGTCTGCTAAAC	CAGATCCTTCGCGGCCGCCTAAAGAGCAAACTCACA
PCDH-SFB-*TRAF6*-RZ_{1-2}	CAGCGGGAGCCCGGATCCATGAGTCTGCTAAAC	CAGATCCTTCGCGGCCGCCTAAACCTGTCTCCTTGG
PCDH-SFB-*TRAF6*-RZ_{1-3}	CAGCGGGAGCCCGGATCCATGAGTCTGCTAAAC	CAGATCCTTCGCGGCCGCCTATGCCAAAGGACAGTT
PCDH-SFB-*TRAF6*-C	CAGCGGGAGCCCGGATCCACCATTCGAACCCTT	CAGCGGGAGCCCGGATCCATGAGTCTGCTAAAC
pSG5-HA-*REGγ*	GGAATTCATGGAAAAATGGATC	ACTCGAGGTACAGAGTCTCTGC

Table S3. Primers.

Table S4 Cell regent and resource

REAGENT or RESOURCE	SOURCE	IDENTIFIER
**Antibody**		
Mouse-anti-β-Actin antibody	MBL International	Cat# M177-3; RRID:AB_10697039
Rabbit-anti-REGγ antibody	Cell Signaling Technology	Cat# 2412; RRID:AB_10695726
Mouse-anti-REGγ antibody	Proteintech	Cat# 67629-1-Ig; RRID:AB_2882830
Rabbit-anti-TRAF6 antibody	Abcam	Cat# ab33915; RRID:AB_778572
Mouse-anti-TRAF6 antibody	Sigma-Aldrich	Cat# SAB1406553; RRID:AB_10758632
Rabbit-anti-NF-κB p65 antibody	Cell Signaling Technology	Cat# 8242; RRID:AB_10859369
Rabbit-anti-Phospho-NF-κB p65 antibody	Cell Signaling Technology	Cat# 3033; RRID:AB_331284
Rabbit-anti-p38 MAPK antibody	Cell Signaling Technology	Cat# 9212; RRID:AB_330713
Rabbit-anti-Phospho-p38 MAPK antibody	Cell Signaling Technology	Cat# 9211; RRID:AB_331641
Rabbit-anti-Erk1/2 antibody	Cell Signaling Technology	Cat# 9102; RRID:AB_330744
Rabbit-anti-Phospho-Erk1/2 antibody	Cell Signaling Technology	Cat# 9101; RRID:AB_331646
Rabbit-anti-JNK antibody	Cell Signaling Technology	Cat# 9252; RRID:AB_2250373
Rabbit-anti-Phospho-JNK antibody	Cell Signaling Technology	Cat# 9251; RRID:AB_331659
Rabbit-anti-HA-tag antibody	Proteintech	Cat# 51064-2-AP; RRID:AB_11042321
Mouse-anti-DDDDK-tag antibody	MBL International	Cat# M185-3L; RRID:AB_11123930
Alexa Fluor 680-AffiniPure Goat Anti-Mouse IgG (H+L)	Jackson ImmunoResearch Labs	Cat# 115-625-146; RRID:AB_2338935
Alexa Fluor 790-AffiniPure Goat Anti-Mouse IgG (H+L)	Jackson ImmunoResearch Labs	Cat# 115-655-146; RRID:AB_2338944
Alexa Fluor 790-AffiniPure Goat Anti-Rabbit IgG (H+L)	Jackson ImmunoResearch Labs	Cat# 111-655-144; RRID:AB_2338086
Peroxidase-AffiniPure Goat Anti-Mouse IgG (H + L)	Jackson ImmunoResearch Labs	Cat# 115-035-003; RRID:AB_10015289
Peroxidase-AffiniPure Goat Anti-Rabbit IgG (H + L)	Jackson ImmunoResearch Labs	Cat# 111-035-003; RRID:AB_2313567
Alexa Fluor® 488-AffiniPure Goat Anti-Mouse IgG (H+L)	Jackson ImmunoResearch Labs	Cat# 115-545-146; RRID:AB_2307324
Alexa Fluor 594-AffiniPure Goat Anti-Rabbit IgG (H+L)	Jackson ImmunoResearch Labs	Cat# 111-585-003; RRID:AB_2338059
Rabbit-anti-NIP30 antibody	Proteintech	Cat# 16830-1-AP; RRID:AB_2109703
p-NIP30	This paper	N/A
Experimental models: Cell lines		

HEK293T cell	ATCC	Cat# CRL-3216; RRID: CVCL_0063
BMMs	This paper	N/A
Osteoclasts	This paper	N/A
MSCs	This paper	N/A
Osteoblasts	This paper	N/A
Experimental models: Organisms/strains		
Mouse: C57BL/6J	Jackson Laboratories	Cat# JAX 000664; RRID: IMSR_JAX:000664
Mouse: *REGγ* KO	a kind gift from Dr. John J. Monaco	N/A
Mouse: *REGγ*$^{fl/fl}$	Zhu et al.	N/A
Mouse: LSL-*REGγ*	Tu et al.	N/A
Mouse: *LysM*-Cre	Clausen et al.	N/A
Mouse: *Traf6*$^{fl/fl}$	This paper	N/A
Mouse: *Nip30*$^{4A/4A}$	This paper	N/A
Biological samples		
Human OP and non-OP samples	Shanghai General Hospital	N/A
Human serum samples	Shanghai General	N/A
Chemicals, peptides, and recombinant proteins		
Recombinant Mouse M-CSF Protein	R&D Systems	416-mL-050
Recombinant Mouse	R&D Systems	462-TEC-010/CF
Dexamethasone	Sigma-Aldrich	D4902
β-Glycerophosphate disodium salt hydrate	Sigma-Aldrich	G6376
Sodium L-ascorbate	Sigma-Aldrich	A4034
C25-140	MedChemExpress	HY-120934
TTP22	MedChemExpress	HY-15479
Silmitasertib (Synonyms: CX-4945)	MedChemExpress	HY-50855
Bone slices	Immunodiagnostic Systems	DT-1BON1000-96
Versene	gibco	15040066
FBS	gibco	10099-141
Australia Origin Fetal Bovine Serum	Avantor Seradigm	76294-180
MEM Alpha basic	gibco	C12571500BT
Critical commercial assays		
Acid Phosphatase, Leukocyte (TRAP) Kit	Sigma-Aldrich	387A
Human TNF Receptor Associated Factor 6 (TRAF6) ELISA Kit	Abbexa	abx153370-96tests
BCIP/NBT Alkaline Phosphatase Color Development Kit	Beyotime	C3206
Alizarin Red S Kit	Beyotime	C0140
PSME3 mouse ELISA kit	Hengyuan Biotechnology	HS1411-Mu
PSME3 human ELISA kit	Hengyuan Biotechnology	HB3546-Hu
CTX-1 human ELISA kit	Hengyuan Biotechnology	HB2390-Hu

P1NP human ELISA kit	Hengyuan Biotechnology	HB2234-Hu
Oligonucleotides		
Primers for qRT-PCR, sequence provided inTable S3	This paper	N/A
Primers for vector constructions, sequence provided inTable S3	This paper	N/A
Primers for Genotype identification, sequence provided inTable S3	This paper	N/A
Recombinant DNA		
Plasmid:PCDH-SFB-TRAF6 WT and mutations	This paper	N/A
Plasmid:pSG5-HA-REGγ	This paper	N/A
Software and algorithms		
ImageJ (version 1.8.0)	National Institutes of Health(NIH)	https://imagej.net/Welcome
GraphPad Prism 8.0	GraphPad Software Inc.	https://www.graphpad.com/
Adobe Illustrator	Adobe Illustrator (Ai)	https://www.adobe.com/products/illustrator.htm
OsteoMeasure Analysis System	Osteometrics	http://www.osteometrics.com/
Skyscan NRecon software	Bruker	N/A
CTAnalyser software	Bruker	N/A
CTVox software	Bruker	N/A
Image Studio Software	LI-COR Biotechnology	https://www.licor.com/bio/empiria-studio/

Table S4. Cell regent and resource.
